# Prevalence of mental health in relation to religious practices in a contemporary small-scale older Amdo Tibetans

**DOI:** 10.1371/journal.pmen.0000099

**Published:** 2025-01-02

**Authors:** Liqiong Zhou, Aijie Zhang, Yasi Zhang, Yuan Chen, Erhao Ge, Juan Du, Zhao Hu, Ruth Mace, Yiqiang Zhan

**Affiliations:** 1 Department of Epidemiology, School of Public Health (Shenzhen), Sun Yat-Sen University, Shenzhen, China; 2 Institute of Environmental Medicine, Karolinska Institute, Unit of Integrative Epidemiology, Stockholm, Sweden; 3 Department of Anthropology, University College London, London, United Kingdom; 4 State Key Laboratory of Grassland Agro-Ecosystem, College of Ecology, Lanzhou University, Lanzhou, China; 5 Institute for Advanced Study in Toulouse, School of Economics, Toulouse, France; IISER Bhopal: Indian Institute of Science Education and Research Bhopal, INDIA

## Abstract

Amdo Tibetans, a devout and cohesive non-industrial society, intertwine religious practices, such as pray, kowtow, and pilgrimage, aspect of their private and public lives. This unique cultural and religious framework fosters a profound connection between their religious practices and health behaviors. The primary aim of this study was to assess the links between religious practices (pray, kowtow, and pilgrimage) and mental health, as well as physical activity and physical function among older Amdo Tibetans. A cross-sectional field survey was conducted involving 538 agropastoral older Amdo Tibetans aged ≥40, situated in the eastern Tibetan Plateau. Evaluated various mental health scales, including psychological well-being (Resilience (SRS-7) and Flourish (SFI-12)), distress (Depression (CESD-8) and Anxiety (GAD-7), and social integration (F_SozU K-6), were assessed, along with objectively measured parameters such as moderate-vigorous physical activity (MVPA), sleep duration, grip strength, gait speed, and walking endurance. Multivariable linear regression models were used to assess the associations. Bonferroni correction was applied for multiple testing, adjusting for socio-demographic characteristics, health status, and health behavior. The study revealed significant positive associations between participation in religious practices and psychological well-being, social integration, and self-rated health, with inverse associations observed with psychological distress. MVPA was positively correlated with daily kowtow. Daily kowtow also showed positive associations with sleep duration. In terms of physical function, daily prayer was negatively associated with grip strength, while daily kowtow exhibited positive correlations with grip strength and gait speed, but distant pilgrimage showed no significant. Religious practices among older Amdo Tibetans were associated with positive mental health, while presenting complex and contrasting effects on physical activity and functional health. In contrast to industrialized societies, unique Amdo Tibetan culture provides a distinctive lens for exploring the relationship between religion and health.

## Introduction

Religiosity serves as a crucial determinant of public health outcomes across diverse populations [1]. Religious practices, while demanding significant investments of time, energy, and resources, provide documented benefits including emotional regulation, positive affect enhancement, and social integration [2,3]. Religious ritualistic practices, including daily prayer, daily kowtow, and distant pilgrimage were in some way derived from established traditions that developed over time within a community [4]. Skirbekk et al. [5] predict that the proportion of religious believers over 60 years old will be nearly 32% in 2050, making them the oldest religious group. This indicates that older adults are assuming a more significant role in the global religious population.

While the growing prominence of religious older adults is evident, the health implications of religious practices within specific ethnic populations remain inadequately explored. Existing research predominantly focuses on special racial groups and age ranges, particularly adolescents and young adults. For example, studies indicated that participation in religious practices protects Iranian university students from engaging in risky behaviors, while religious well-being also alleviates perceived fear of COVID-19 [6,7]. However, it is unclear whether the potential health benefits of religious practices apply to older adults or the specific context of Iranian students, highlighting the need for further research in different societies.

In secular, industrialized societies, regular religious attendance has been linked to a wide range of favorable health outcomes, including lower mortality rates, reduced risk of depression, lower chronic disease burden, and improved self-rated physical health [8–11]. For instance, studies in Marin County, California, have demonstrated that individuals who regularly attend religious services experience lower mortality rates, suicide rates, and depression while regular attendees in Denmark exhibit lower hospitalization rates [9,11]. However, these findings predominantly reflect the experiences of industrialized populations, potentially overlooking the unique cultural and environmental factors that shape religious practices and health outcomes in non-industrialized societies. In non-industrialized societies like that of the Amdo Tibetans, their strong adherence to Gelugpa Buddhism and the integration of religious practices into daily life offer a unique context to explore the link between religion and health. Religious practices are not only central to social and communal life but also intricately tied to both physical and mental health behaviors. Tibetan Buddhist practices such as daily prayer and kowtow require physical exertion, which contributes to their overall health. In addition, the spiritual and social aspects of these practices provide emotional and psychological benefits [2,3]. In industrialized societies, religious practices and daily life may be more compartmentalized. However, religious practices are fully integrated into the daily life of Amdo Tibetans, offering a unique perspective on the relationship between religious practices and health.

WHO recommends that older adults engage in at least 150 minutes of moderate to vigorous aerobic physical activity weekly [12]. Physical inactivity ranks as the fourth leading risk factor for human mortality, following high blood pressure, tobacco use, and high blood sugar [13]. Accurate and valid measurement of physical activity is crucial for health epidemiology, and objective assessment in both laboratory and free-living environments is facilitated by devices [14,15]. To our knowledge, some evidence on the relationship between religion and health, most studies have focused on a limited range of mental health outcomes. Given the broad definition of health as “a state of complete physical, mental, and social well-being”, incorporating physical activity into research on religion and health is essential. Our population, living a subsistence-based lifestyle characterized by high levels of obligatory physical activity, religious beliefs, and ritual activities, often lacks access to medical care, with many individuals never having their blood pressure measured until our study. This presents an intriguing contrast to industrialized populations [15,16]. Additionally, most previous studies of physical activity have relied on self-reporting. While data obtained from questionnaires can be effective, participants frequently overlook light activity, and their physical, cognitive, and emotional conditions can influence reporting accuracy [17]. To address this, we used wearable activity trackers to provide more objective information.

Amdo Tibetan society, a non-industrial community rooted Tibetan Buddhism, is characterized by a subsistence economy, strong kinship ties, and deep-rooted religious traditions. In this context, we aimed to investigate the association between religious practices (pray, kowtow, and pilgrimage) and physical and mental health among older Amdo Tibetans. We hypothesized that significant positive associations between participation in religious practices and psychological well-being, physical activity and function, with inverse associations observed with psychological distress. This investigation contributes to filling a knowledge gap in the health implications of religious practices within a culturally unique, understudied population, offering insights distinct from those observed in industrialized societies.

## Methods

### Study design, sample size, and sampling method

**Fig 1:**
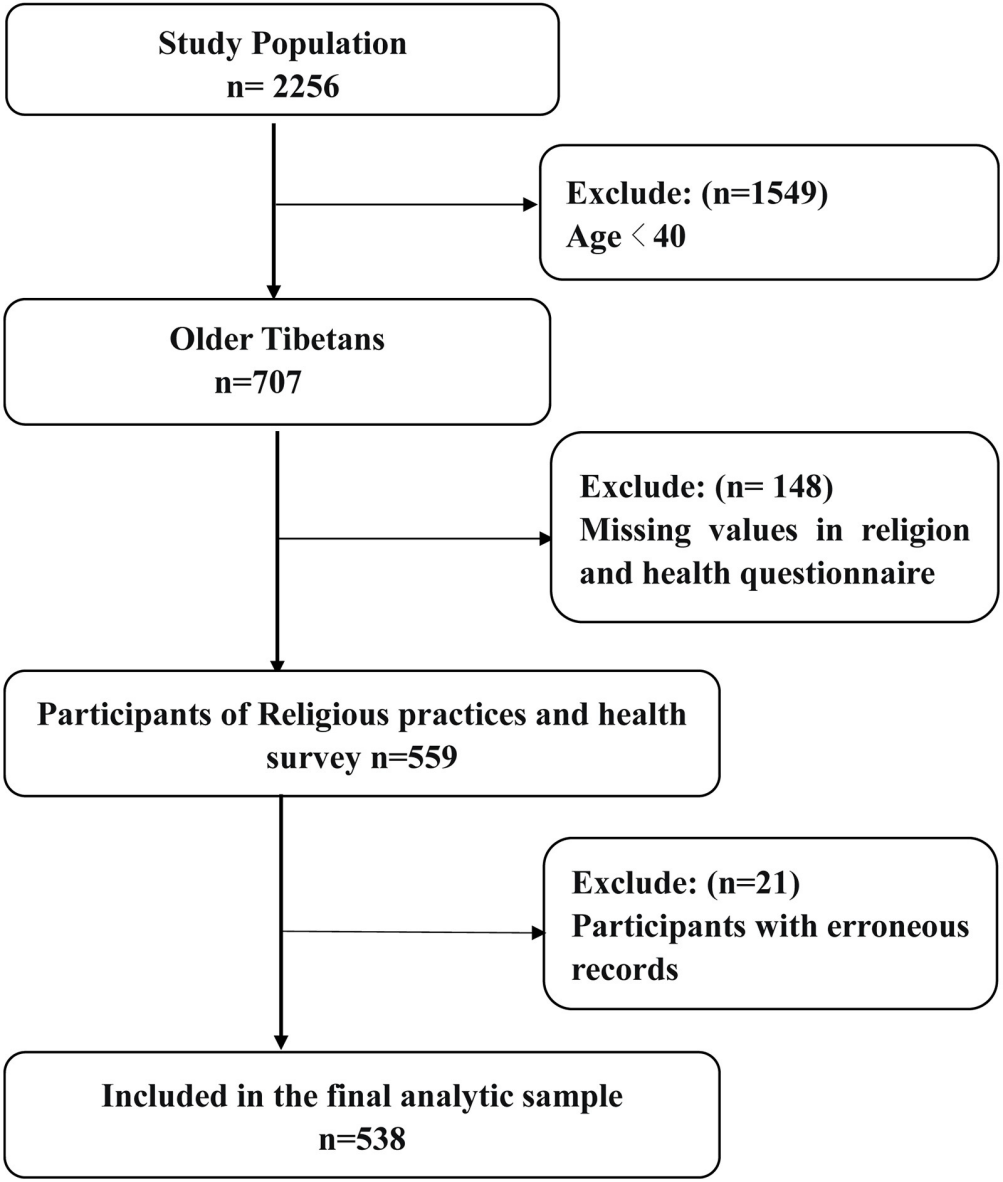
Flow diagram of sample derivation in the study population.

We conducted a cross-sectional field survey from May 21 to July 26, 2023, in a county located in the eastern Tibetan Plateau, home to agropastoral Amdo (Tibetan: ཨ༌མདོ; Chinese: 安多) Tibetans in Gansu Province, China. Initially, we recruited 2,256 participants from 328 households across 15 villages, excluding those with a history of mental health issues. Further exclusions comprised individuals aged under 40 (N = 1,549), those with missing data in the religion and health questionnaire (N = 135), participants with erroneous records (N = 21), and 13 people who did not complete the kowtow questionnaire, resulting in a final sample of 538 individuals **(**Fig 1). This region is characterized by high-altitude landscapes, with an average elevation of over 3,000 meters above sea level, harsh environmental conditions, cold winters, and limited agricultural seasons. The main livelihood of the population is agropastoralism, combining subsistence agriculture with livestock herding, particularly yaks and sheep, which are the most important components of the economy. Amdo Tibetans indiscriminately believe in the denomination of Gelug (Yellow Hats), one of the main branches of Tibetan Buddhism [16]. Pervasive religious practices occupy a great deal of residential life, including the following three practices: pray (pray is usually performed with rosary beads while counting simultaneously), kowtow (prostrating to Buddha images at home), and pilgrimage (making long distant pilgrimage to famous monasteries located all over the Tibetan plateau) [4,18] (Fig A in S1 Text). To facilitate the survey, we employed cluster sampling to conduct the survey, integrating household questionnaires with wearable wristband data collection, a method chosen for its effectiveness in geographically challenging environments, enabling efficient surveys of entire households in selected villages and facilitating comprehensive field surveys and family interviews. Each interview, lasting approximately 2 hours, involved local interpreters to assist with communication, especially for those who spoke only Tibetan. Additionally, all investigators were trained prior to the survey and learned how to use survey tools. Strict measures of questionnaire quality control, data entry and logical examination of results were implemented throughout the survey.

### Ethical approval

This project, granted approval by the Medical Ethics Committee of the School of Public Health of Sun Yat-sen University (Shenzhen) (No. 2023030), obtained informed consent at three levels: (1) Local Amdo Tibetans administration that oversees research projects and given written consent, (2) verbal consent from village leadership, and (3) study participants.

### Assessment of religious practices

Participation in religious practices was determined based on responses to the following questions: "Do you participate in daily prayer practices?", "Do you participate in daily kowtow?", and "Do you participate in distant pilgrimage?". Responses were categorized as either ’yes’ or ’no’.

### Assessment of mental health

Mental health status was measured using self-reported questionnaire scales. Psychological well-being was assessed through psychological resilience (SRS-7) [19], secure flourishing index (SFI-12) [20], optimism [21,22], and mastery [23]. Psychological distress was evaluated using measures of depression (CESD-8) [24], anxiety (GAD-7) [25], and hopelessness [26,27]. Social integrations were evaluated using the social support scale (F-SozU K-6) [28] and marital satisfaction scale (C-KMS-3) [29]. Additionally, self-rated health (SRH) [30] was included as another indicator. Detailed descriptions of each mental health scale and the corresponding Cronbach’s alpha coefficients are provided in Table A in S1 Text. All scales have been previously validated and demonstrated good reliability within this sample.

### Assessment of physical activities

Participants were distributed accelerometers (Redmi band 2) to wear 24 hours per day on the dominant hand for a requested 1–2 days and were asked not to engage in religious practices while wearing the accelerometer in a free-living environment. Briefly, the Redmi band 2 is a fitness tracker that uses a three-axis accelerometer to detect any motion or movement and then converts it into measurable data [15]. Time spent in MVPA was recorded when the heart rate reached 65% of the maximum or the cadence reached 100 beats/min during exercise. Objective sleep duration per day was also measured using an accelerometer. Exclusion criteria were participants who continuously removed their accelerometers or had less than one day of data.

### Assessment of physical functions

Grip strength is frequently used to assess muscle strength in industrialized contexts. A Camry digital hand dynamometer (HUWAIREN, Guangdong) was used to measure grip strength. Participants used their dominant hand to squeeze the dynamometer as hard as possible in three grip strength trials, and the highest recorded value among the trials was documented as the maximum grip strength (kg), following the protocol of the U.S. National Health and Nutrition Examination Survey [31].

Gait speed, a valid indicator linked to incident disability, dementia, mortality, and other adverse outcomes, was considered a "sixth vital sign" and is recommended by the NIH Toolbox for functional vital sign assessments in older Adults [32,33]. Participants were instructed to walk at their usual pace, starting from a standing position. Gait speed was calculated for each participant based on a 4-meter walk distance, using the formula: speed = distance (in meters) / time (in seconds).

Walk tests that record distance travelled during a set time period are often used to assess walking endurance in industrialized populations. In this study, we assessed walking endurance using a 2 minutes’ walk test [34]. A flat 4m walkway was cleared and marked at each field site for a walking test. Participants were instructed to walk as fast as possible for 2 minutes, and researchers tallied the laps. At the end of the test, researchers measured the total distance traveled.

### Assessment of covariates

Socio-demographic: age (years), sex (female, male), number of yaks, annual expenditure (< 20000, 20000–34999, ≥ 35000 *CNY*), number of siblings, marital status (unmarried, married, widow/widower), education attainment (illiteracy, literacy), household size, distance to town.

Health status: systolic blood pressure (< 140, ≥ 140), body mass index (calculated as weight in kilograms divided by height in meters squared) (< 25, ≥ 25), history of arthritis (yes or no), heart disease (yes or no), chronic bronchitis (yes or no), insomnia (yes or no). Medical conditions were identified by asking participants if they had been diagnosed with arthritis, heart disease, insomnia and chronic bronchitis by hospitals at or above the county level. Systolic blood pressure (SBP) was measured by an electronic sphygmomanometer (Taibang, Model W104L) after the participants remaining relaxed for 5 min. The body mass index (BMI) was calculated by dividing an individual’s weight in kilograms by the square of their height in meters (kg/m2).

Health behavior: smoking status (never, former, current), drinking status (never, former, current).

### Statistical analysis

All statistical analyses were performed with R 4.2.2. Participant characteristics were summarized across participants and non-participants in three religious practices (pray, kowtow and pilgrimage) using means and standard deviations for continuous variables and percentages for categorical variables (Table 1). The statistical difference in the distribution of the mental health scores and device measurements of physical activity and function across different religious practices were assessed using One-way analysis of variance (ANOVA). 45 independent separate multivariate regression models (15 physical and mental health variables * 3 models) were built to further explore the relationship between participants’ religious practices and indicators of physical and mental health. Consequently, we present our results in summarized form within tables. We briefly discuss findings of models where each variable was tested separately. Before conducting the analysis, we assessed variance inflation factors (VIFs) to ensure the absence of multicollinearity when incorporating multiple measures in regression models. Our finding indicated generally low VIF values (mean VIF = 1.26), with none surpassing the conservative cutoff of 5 [35]. The analysis proceeded in a series of regression steps where main effects were entered in the first model without any covariates (Model 1). Subsequently, we continued assess the effects of religious practices on mental health, physical activity, and physical function while adjusting for age, sex, number of yaks, annual expenditure, number of siblings, marital status, education attainment, household size, distance to town, SBP, BMI, history of arthritis, heart disease, chronic bronchitis, insomnia (Model 2). Finally, adjustments for health behavior, smoking status and drinking status (never, former, current) (Model 3). BMI data was missing from 55 participants for whom no weight data was available. Multiple imputation (with 5 imputed data sets) was performed to impute missing data. Bonferroni correction was used to correct for multiple testing. All statistical tests were two-tailed and a P < 0.05 was considered statistically significant. This study is reported as per the Strengthening the Reporting of Observational Studies in Epidemiology (STROBE) guideline (S1 Checklist).

**Table 1:** Characteristics of sample characteristics according to the different types of religious practices among older Amdo Tibetans. (n = 538).

Characteristic		Pray		Kowtow		Pilgrimage	
	Overall N = 538	Non-participation N = 271	Participation N = 511	Non-participation N = 189	Participation N = 349	Non-participation N = 255	Participation N = 283
Social demographic:							
Age	66.10 (12.04)	60.89 (15.59)	66.38 (11.78)	66.20 (13.22)	66.05 (11.37)	64.11 (12.03)	67.89 (11.78)
Sex							
Female	329 (61%)	11 (41%)	318 (62%)	92 (49%)	237 (68%)	161 (63%)	168 (59%)
Male	209 (39%)	16 (59%)	193 (38%)	97 (51%)	112 (32%)	94 (37%)	115 (41%)
Education attainment							
Illiteracy	407 (76%)	17 (63%)	390 (76%)	143 (76%)	264 (76%)	195 (76%)	212 (75%)
Literacy	131 (24%)	10 (37%)	121 (24%)	46 (24%)	85 (24%)	60 (24%)	71 (25%)
Household size	7.11 (2.45)	7.22 (1.63)	7.12 (2.50)	7.16 (2.44)	7.08 (2.45)	6.92 (2.41)	7.31 (2.50)
Marital status							
Unmarried	11 (2.0%)	0 (0%)	11 (2.2%)	4 (2.1%)	7 (2.0%)	5 (2.0%)	6 (2.1%)
Married	466 (87%)	25 (93%)	441 (86%)	164 (87%)	302 (87%)	227 (89%)	239 (84%)
Widow/widower	61 (11%)	2 (7.4%)	59 (12%)	2 (7.4%)	7 (2.0%)	5 (2.0%)	6 (2.1%)
No. Yaks	36.25 (38.62)	39.33 (48.32)	36.09 (38.09)	30.16 (33.66)	39.56 (40.72)	34.70 (35.66)	37.66 (41.11)
Annual expenditure							
≤19999	169 (31%)	16 (59%)	153 (30%)	61 (32%)	108 (31%)	74 (29%)	95 (34%)
20000–34999	216 (40%)	7 (26%)	209 (41%)	60 (32%)	156 (45%)	107 (42%)	109 (39%)
≥35000	153 (28%)	4 (15%)	149 (29%)	68 (36%)	85 (24%)	74 (29%)	79 (28%)
No. Siblings	2.22 (2.15)	2.37 (1.64)	2.22 (2.18)	2.23 (2.25)	2.22 (2.10)	1.99 (1.89)	2.43 (2.35)
Distance to town	70.05 (24.19)	70.15 (25.64)	70.04 (24.13)	69.89 (24.41)	70.13 (24.10)	69.51 (24.28)	70.53 (24.14)
Religious practices:							
Pray time (min)		0.00 (0.00)	109.59 (86.07)				
Kowtow time (min)				0.00 (0.00)	34.31 (30.18)		
Pilgrimage (day)						0.00 (0.00)	37.68 (60.53)
Mental Health:							
Psychological Well-Being							
Resilience (range:0–22)	15.74 (3.05)	15.37 (3.18)	15.76 (3.05)	15.41 (3.03)	15.93 (3.05)	16.13 (3.05)	15.40 (3.01)
Flourish (range:0–120)	81.39 (11.37)	75.70 (13.36)	81.69 (11.19)	79.13 (11.00)	82.61 (11.40)	80.22 (11.18)	82.44 (11.46)
Optimism (range:0–12)	7.71 (1.57)	6.96 (1.68)	7.75 (1.56)	7.31 (1.60)	7.93 (1.51)	7.61 (1.36)	7.80 (1.74)
Mastery (range:0–8)	4.63 (1.47)	4.96 (1.63)	4.61 (1.46)	4.54 (1.58)	4.67 (1.41)	4.70 (1.40)	4.56 (1.53)
Psychological Distress							
Depression (range:0–8)	2.78 (2.18)	2.74 (1.99)	2.78 (2.19)	3.11 (2.04)	2.60 (2.24)	2.86 (2.13)	2.71 (2.23)
Anxiety (range:0–21)	2.84 (3.85)	5.26 (4.93)	2.72 (3.75)	3.01 (3.96)	2.76 (3.79)	2.80 (3.63)	2.88 (4.04)
Hopeless (range:0–8)	5.08 (1.45)	4.78 (1.50)	5.09 (1.44)	5.34 (1.29)	4.94 (1.51)	5.24 (1.33)	4.94 (1.53)
Social integration							
Social support (range:0–30)	24.84 (4.14)	22.44 (4.14)	24.97 (4.11)	24.78 (3.65)	24.87 (4.39)	25.11 (3.89)	24.60 (4.34)
Marital satisfaction (range:0–18)	14.38 (1.60)	13.74 (2.09)	14.41 (1.56)	14.58 (1.41)	14.27 (1.68)	14.49 (1.57)	14.28 (1.62)
Other factor							
Self-related health (range:0–4)	2.52 (1.15)	2.78 (1.15)	2.51 (1.15)	2.23 (1.34)	2.68 (1.01)	2.50 (1.22)	2.54 (1.09)
Physical activity and function:							
MVPA (h/day)	3.28 (1.91)	4.83 (1.99)	3.20 (1.88)	3.06 (2.05)	3.41 (1.81)	3.56 (1.85)	3.03 (1.93)
Sleep duration (h/day)	7.84 (1.19)	7.74 (1.12)	7.84 (1.20)	7.66 (1.14)	7.93 (1.21)	7.73 (1.17)	7.92 (1.21)
Grip strength (Kg)	20.79 (10.70)	28.76 (12.12)	20.38 (10.47)	21.65 (11.93)	20.34 (9.99)	21.20 (11.86)	20.42 (9.55)
Gait speed (4m) (m/s)	0.86 (0.25)	1.06 (0.20)	0.85 (0.25)	0.86 (0.25)	0.87 (0.25)	0.86 (0.23)	0.87 (0.26)
Endurance (2min) (m)	90.68 (23.82)	106.65 (31.03)	90.08 (23.14)	93.34 (28.29)	89.75 (21.39)	92.54 (24.02)	89.42 (23.58)
BMI (kg/m2)							
< 25	256 (53%)	10 (43%)	246 (53%)	83 (54%)	173 (53%)	127 (56%)	129 (50%)
> = 25	227 (47%)	13 (57%)	214 (47%)	71 (46%)	156 (47%)	99 (44%)	128 (50%)
SBP (mmHg)							
< 140	397 (74%)	24 (89%)	373 (73%)	140 (74%)	257 (74%)	205 (80%)	192 (68%)
> = 140	139 (26%)	3 (11%)	136 (27%)	48 (26%)	91 (26%)	50 (20%)	89 (32%)
Disease:							
Arthritis	123 (23%)	7 (26%)	116 (23%)	38 (20%)	85 (24%)	51 (20%)	72 (25%)
Heart disease	79 (15%)	2 (7.4%)	77 (15%)	30 (16%)	49 (14%)	43 (17%)	36 (13%)
Chronic bronchitis	73 (14%)	1 (3.7%)	72 (14%)	25 (13%)	48 (14%)	37 (15%)	36 (13%)
Insomnia	165 (31%)	8 (30%)	157 (31%)	61 (32%)	104 (30%)	84 (33%)	81 (29%)
Health behavior:							
Smoke Status							
Never	469 (87%)	17 (63%)	452 (88%)	148 (78%)	321 (92%)	224 (88%)	245 (87%)
Past	40 (7.4%)	3 (11%)	37 (7.2%)	24 (13%)	16 (4.6%)	16 (6.3%)	24 (8.5%)
Current	29 (5.4%)	7 (26%)	22 (4.3%)	17 (9.0%)	12 (3.4%)	15 (5.9%)	14 (4.9%)
Drink Status							
Never	484 (90%)	24 (89%)	460 (90%)	161 (85%)	323 (93%)	237 (93%)	247 (87%)
Past	42 (7.8%)	2 (7.4%)	40 (7.8%)	22 (12%)	20 (5.7%)	13 (5.1%)	29 (10%)
Current	12 (2.2%)	1 (3.7%)	11 (2.2%)	6 (3.2%)	6 (1.7%)	5 (2.0%)	7 (2.5%)

^1^ Mean (SD); n (%).

SRS: Psychological resilience, flourish, optimism, mastery, CESD: Depression, GAD: Anxiety, hopeless, F_SozU: Social support scale, C_KMS: Marital satisfaction, SRH: Self-related health, BMI: Body mass index, SBP: Systolic blood pressure, MVPA: Moderate-to-vigorous physical activity duration per day.

## Results

### Characteristics of the study participants

**Fig 2:**
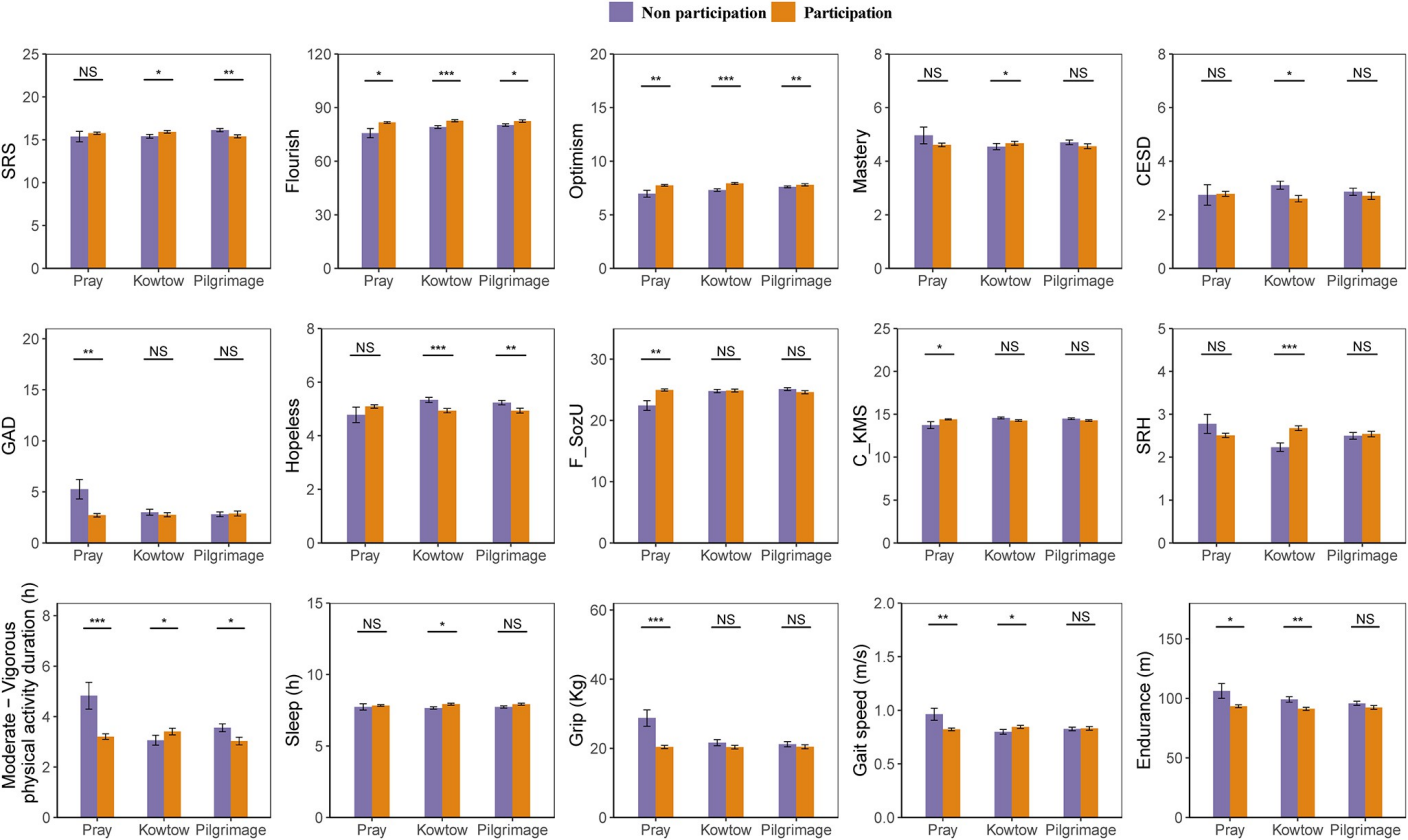
Relationship between participation and non-participation in religious practices and mental health, physical activity, and physical functioning among older Amdo Tibetans. Religious practices: Pray, kowtow, and pilgrimage; Mental health: Psychological well-being (psychological resilience, flourishing, optimism, mastery), psychological distress (depression, anxiety, hopelessness), social integration (social support), marital satisfaction, and self- related health; Physical activity: Moderate-to-vigorous physical activity, sleep duration; Physical function: Grip strength, gait speed, and walking endurance. Orange represents participants, purple represents non-participants. Standard errors are displayed. * p<0.05, ** p<0.01, *** p<0.001.

Descriptive statistics for 538 older Amdo Tibetans show a mean age of 66.10 (12.04) years, with 61.15% being female. Most participants are illiterate (76%) and own an average of 36 yaks per household. Regarding religious practices, 64.87% engage in daily kowtow, 54.46% in distant pilgrimage, and 5.01% do not participate in pray. On average, participants spent 105.18 minutes per day pray, 22.26 minutes per day performing kowtow, and 19.88 days per year on pilgrimage, with females spending significantly more time pray and performing kowtow than males, but no significant gender difference in pilgrimage time (Table 1 and Table B in S1 Text). Kowtow participants exhibited significantly better psychological well-being, self-related health, MVPA, sleep duration, gait speed, and walking endurance than non-participants. However, no significant differences were found in psychological resilience, mastery, hopelessness, self-related health, or sleep duration between those who prayed and those who didn’t. Similarly, there were no significant differences in depression, anxiety, mastery, social support, sleep, grip strength, gait speed, or walking endurance between pilgrims and non-participants (Fig 2).

### Religious practices and mental health

The multiple linear regression model suggested that participation in religious practices was positively associated with psychological well-being, which remained robust after adjusting for covariates in the kowtow group (ie, psychological resilience: β = 0.83, P < 0.01; flourish: β = 3.22, P < 0.01, optimism: β = 0.61, P < 0.001; mastery: β = 0.37, P<0.05), while flourish, optimism, and mastery showing null associations with the pray and pilgrimage groups. However, psychological resilience and pilgrimage participation consistently maintained a negative correlation (β = -0.64, P < 0.05) (Table 2, Table C—F and Table R in S1 Text).

**Table 2:** Multiple linear regression analyses of religious practices (pray, kowtow, and pilgrimage) on mental health (psychological well-being, psychological distress, social integration, and self-rated health), physical activity (moderate-to-vigorous physical activity, sleep duration), and physical function (grip strength, gait speed, and walking endurance) among older Amdo Tibetans. (n = 538).

Non-participation			Religious practices								
			Beta/ P value								
			Pray			Kowtow			Pilgrimage		
			Model 1	Model 2	Model 3	Model 1	Model 2	Model 3	Model 1	Model 2	Model 3
Psychological Well-Being											
Resilience	1[Reference]	N = 538	0.26	0.20	0.41	**0.65** **	**0.85** **	**0.83** **	**-0.84** **	**-0.66** **	**-0.64** **
Flourish	1[Reference]	N = 538	**4.73** **	3.09	3.60	**2.83** ***	**3.22** **	**3.27** **	**1.65** *	1.49	1.54
Optimism	1[Reference]	N = 538	**0.57** **	0.39	0.34	**0.56** ***	**0.61** ***	**0.61** ***	**0.08** **	0.15	0.15
Mastery	1[Reference]	N = 538	-0.40	-0.39	-0.36	**0.19** *	**0.33** **	**0.37** **	-0.16	-0.23	-0.24
Psychological Distress											
Depression	1[Reference]	N = 538	0.24	0.32	0.25	**-0.51** **	**-0.46** **	**-0.44** **	-0.08	-0.16	-0.18
Anxiety	1[Reference]	N = 538	**-2.53** **	**-2.20** **	**-2.33** **	-0.08	-0.24	-0.22	0.15	-0.00	-0.06
Hopelessness	1[Reference]	N = 538	0.49	0.41	0.40	**-0.40** **	**-0.34** **	**-0.33** **	**-0.25** **	**-0.37** **	**-0.37** **
Social integration											
Social support	1[Reference]	N = 538	**2.60** **	**1.74** **	**2.16** **	-0.01	0.16	0.14	-0.57	-0.21	-0.17
Marital satisfaction	1[Reference]	N = 538	**0.78** **	0.57	**0.70** **	-0.28	-0.19	-0.18	-0.14	-0.12	-0.11
Other factor											
Self-related health	1[Reference]	N = 538	-0.45	-0.47	-0.41	**0.48** ***	**0.53** ***	**0.54** ***	-0.02	-0.06	-0.07
Physical activity											
Moderate & vigorous duration	1[Reference]	N = 302	**-1.83** ***	**-1.35** **	**-1.36** **	**0.59** **	**0.42** **	**0.44** **	**-0.54** **	**-0.51** **	**-0.52** **
Sleep duration	1[Reference]	N = 302	-0.01	-0.01	0.01	**0.24** **	**0.34** **	**0.32** **	0.15	0.02	0.01
Physical functions											
Grip strength	1[Reference]	N = 538	**-7.73** ***	**-3.88** **	**-3.09** **	-0.22	**1.64** *	**1.87** **	-0.49	-0.37	-0.47
Gait speed	1[Reference]	N = 538	**-0.16** **	-0.06	-0.07	**0.06** **	**0.07** **	**0.08** *	0.00	0.02	0.02
Endurance	1[Reference]	N = 538	**-10.08** **	-5.72	-4.30	**-6.73** **	**-5.46** **	**-4.93** **	-2.16	-1.63	-1.76

* p<0.05

** p<0.01

*** p<0.001, Significants are marked in bold.

^a^ Model 1: No covariates.

^b^ Model 2 model controlled for age (years),sex (female, male), number of yaks, annual expenditure (<20000, 20000–34999, ≥35000), number of siblings, marital status (unmarried, married, widow/widower), education attainment (illiteracy, literacy), household size, distance to town, systolic blood pressure(<140, ≥140), body mass index (calculated as weight in kilograms divided by height in meters squared) (<25, ≥25), history of arthritis (yes or no), heart disease (yes or no), chronic bronchitis (yes or no), insomnia (yes or no).

^c^ Model 3 included further adjustments for health behavior: Smoking status (never, former, current), drinking status (never, former, current).

For psychological distress, people who participate in kowtow have inversely correlated with depression (β = -0.44, P < 0.05) and hopelessness (β = -0.33, P < 0.05) scores, anxiety (β = -2.33, P < 0.01) showed a negative correlation with prayer, and hopelessness (β = -0.37, P < 0.01) was negatively correlated with pilgrimage participation when adjusting for covariates (Table 2, Table G—I and Table R in S1 Text).

People who participated in pray showed a consistent positive correlation with social support (β = 2.16, P < 0.01) and marital satisfaction (β = 0.70, P < 0.05). Simultaneously, participation in kowtow was also positively associated with self-related health scores (β = 0.54, P < 0.001) (Table 2, Table J—L and Table R in S1 Text).

### Religious practices and physical activity

Average daily time spent in MVPA for 302 participants was 3.19 ± 2.12 hours per day for males (n = 180) and 3.34 ± 1.76 hours per day for females (n = 122) and the average sleep duration was 7.84 ± 1.19 hours (Table B in S1 Text). Participation in kowtow was significantly positively associated with time spent in MVPA (β = 0.44, P < 0.05) compared to non-participants, whereas participation in pray (β = -1.36, P <0.05) and pilgrimage (β = -0.52, P <0.05) showed a significant negative association with time spent in MVPA (Table 2). Results did not change when covariates were included in the full model (Table M and Table R in S1 Text). Compared to non-participants, individuals who engaged in kowtow group (β = 0.32, P <0.01) demonstrated a significantly positive association with sleep duration, while this association was not significant in the pray and pilgrimage groups (Table 2, Table N and Table R in S1 Text).

### Religious practices and physical function

**Fig 3:**
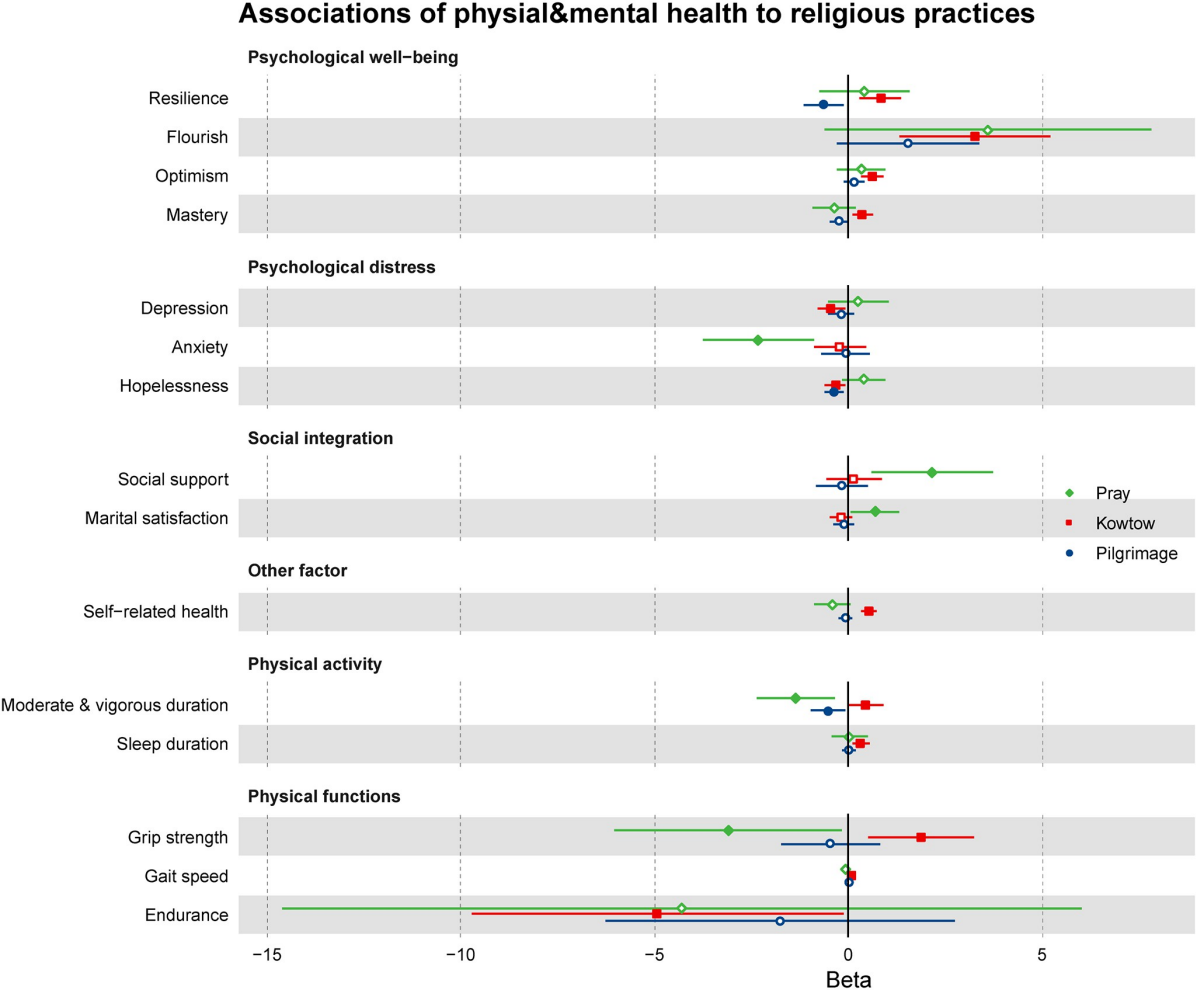
Forest plot of full model after Bonferroni correction. Beta show physical and mental health vary with different religious practices pattern for older Amdo Tibetans. Bonferroni adjusted P Value < 0.05/15 = 0.003 (two-sided). Significant points are solid, while insignificant points are hollow.

The average grip strength of older Amdo Tibetans is 20.79 ± 10.7 kg, the walking speed is 0.86 ± 0.25 m/s, and the walking endurance is 90.68 ± 23.91m, respectively (Table 1, Table B in S1 Text). Compared with individuals who never attended religious practices, participants engaging in kowtow showed positive associations with grip strength (β = 1.87, P <0.01) and gait speed (β = 0.08, P <0.05), negative associations with walking endurance (β = -4.93, P <0.05), after adjusting for all covariates. Additionally, participants who prayed were negatively associated with grip strength (β = -3.09, P <0.05). However, participation in pilgrimage was null associated with physical function (Table 2, Table O–P and Table R in S1 Text). To account for all multiple testing, Bonferroni correction was applied in the forest plot (Fig 3).

## Discussion

### Main findings

To our knowledge, this is the first study to explore the relationship of religious practices with mental health, physical activity, and physical function among older Amdo Tibetans. Our findings revealed that religious practices were positively associated with psychological well-being, including psychological resilience, flourishing, optimism, and mastery. While it is negatively associated with psychological distress, such as depression and anxiety. Regarding social integration, our findings indicated that religious practices were positively associated with social support and marital satisfaction. Furthermore, daily kowtow was positively correlated with MVPA, sleep duration, grip strength, and gait speed, while daily prayer was negatively associated with MVPA and grip strength, highlighting the seemingly paradoxical findings regarding the relationship between religious practices and physical health and function.

### Religious practices and mental health

Our findings are consistent with increasing research evidence showing that religion is associated, both cross-sectionally and longitudinally, with improved physical health, better mental health, and longer survival. A meta-analysis conducted between 1983 and 2019 confirmed the positive relationship between religiousness and better mental health [36]. Longitudinal research indicated that weekly religious service attendance predicted greater life satisfaction and positive emotions in adulthood [10]. A cross-sectional survey of 26,678 Danes showed that low health and life satisfaction were negatively associated with religious needs [37]. Similarly, some other cross-sectional survey of Iranians showed that religious well-being can be a strong predictor of stress levels when combined with hope and resilience [38–40]. A 15-year study of 167,577 older Thai adults found that those with religious affiliations had higher MVPA and longer sleep duration than non-religious individuals [41]. Previous studies show that frequent religious practice increases the likelihood of expressing values like gratitude, reputation, generosity, and devotion [4,42].

### Religious practices and physical health

Anthropologically, the link between religiosity and mental health depends on identity integration, current beliefs, practices, motivations, and environmental support for one’s sexual or religious identity [43]. Meanwhile, MVPA is higher than the World Health Organization PA guidelines because the area is dominated by agriculture and animal husbandry. Populations live a subsistence-based lifestyle that necessitates high levels of obligatory PA. The elderly are also one of the main labor forces. The time when we collect data coincides with the busiest autumn harvest season of the year, so PA duration will be higher than at other times. Furthermore, age-related declines in physical function challenge the survival and reproductive success of older individuals, as seen in limited evidence from two East African populations: Hadza hunter-gatherers in Tanzania and Pokot pastoralists in Kenya [44]. Older participants who engage in daily kowtow, a practice involving repetitive body movements, may contribute directly to increased MVPA, thereby enhancing physical function. This may explain the observed positive correlation between religious practices and grip strength and gait speed [45]. Conversely, pilgrimage, which often requires extensive travel and physical exertion, may have intermittent yet intense impacts on physical activity patterns, differing significantly from daily practices like prayer. Additionally, we also noted how certain practices might inversely affect physical activity, such as extended periods of prayer, which could contribute to reduced MVPA by involving long sedentary durations [4].

### Biological mechanisms

From the perspective of neurobiological research, changes in neurotransmitters contribute to improved symptoms of anxiety and depression and may explain, in part, how religious practice mitigates psychogenic [46]. As some studies have shown, spiritual experiences are important in preventing the onset of depression, suggesting a possible mechanism by which cortical thickening is associated with spirituality in certain brain regions [47]. Religious practices also in terms of their inner peace could help improve spiritual care for religious cancer patients [48].

### Contributions

Our study offers several significant contributions. First, the data was collected under free-living conditions, providing a unique "natural laboratory" where participants maintained their daily routines, which is difficult to replicate in laboratory environments. Another strength is that we carefully considered confounding factors in our analysis. Sociodemographic factors, such as older age or those with higher household incomes, who may have more resources and time to participate in religious practices, could bias the observed associations. Health status variables, objective measures such as SBP and BMI, were also considered to strengthen the robustness of our findings. People with arthritis or heart disease may be less likely to engage in vigorous activity, and this approach helped isolate independent associations between religious activity and health outcomes.

### Limitations

Despite the strengths of this study, several limitations should be acknowledged. First, cross-sectional design restricts our ability to establish causal relationships between religious practices and health outcomes. Longitudinal studies are needed to observe changes over time and make more definitive causal inferences. Second, the relatively small sample size and specific focus on the older Tibetans limit the generalizability of our findings to other demographic groups. Future research should include larger, more diverse samples to enhance generalizability. Third, the short-term duration of physical activity measurements may not fully capture participants’ typical activity levels over an extended period. Although we used accelerometers to provide objective data, the brief monitoring period is a limitation. Extending the monitoring period in future studies to a duration of one to two weeks would provide a more comprehensive view of physical activity patterns.

## Conclusions

In summary, our research highlighted the integration of religious practices into health promotion efforts, particularly for mental and physical well-being among older Tibetan populations. We suggested that health policies should consider the cultural significance of religious practices. This approach can inform interventions that align with local values, potentially using religious practices to cope with stress and maintain mental health. Traditional health research often overlooks the experiences of ethnic minorities, contributing to a potential bias of racism. By focusing on Amdo Tibetans, our study provides a unique perspective that challenges this bias and promotes inclusive research methodologies. Future research is needed to further longitudinal and cross-cultural studies to explore causal relationships and examine different forms of religious practices or cultural contexts in other ethnic groups to validate our findings and expand their applicability.

## Supporting information

S1 Checklist STROBE statement—Checklist of items that should be included in reports of *cross-sectional studies*. (DOCX)

S1 Text Supplementary appendix. (DOCX)
