# Supplementary material for: Prevalence of mental health in relation to religious practices in a contemporary small-scale older Amdo Tibetans
Source: PLOS Ment Health. 2025 Jan 2;2(1):e0000099. doi: 10.1371/journal.pmen.0000099 (PMC12798266; doi:10.1371/journal.pmen.0000099)
Supplement: S1 Text — (DOCX) [file pmen.0000099.s002.docx]

**SUPPLEMENTARY APPENDIX**

**Table A.** Assessment of Psychological Questionnaire

| Category | Scale | Abbreviation | Definition of scale |
| --- | --- | --- | --- |
| Psychological Well-Being | Psychological resilience | SRS-7 | **SRS** ^1^**:** The SRS is based on the available data collected through seven questions related to resilience in the CLHLS which is emphasized coping and adjustment among the elderly. It was calculated based on responses to 7 statements: 1. Do you feel the older you get, the more useless you are? 2. Do you always look on the bright side of things? 3. Do you often feel fearful or anxious? 4. Do you often feel lonely and isolated? 5. To whom do you usually talk most frequently in daily life? 6. Who do you ask first for help when you have problems/difficulties? 7. Can you make your own decisions concerning your personal affairs? Items1-4 and 7 were self-reported on 5 points (0: always; 1: Often; 2: Sometimes; 3: Very few; 4: Never), items 5 and 6 were self-reported on 2 points (1. Family members/friends/neighbors/social workers/caregivers; 0. Nobody), items 2 and 7 were reverse coded. We obtained from the survey the total SRS ranges from 0 to 22, with higher scores reflecting greater resilience. The scale shows good reliability in this work (α=0.68) |
|  | Flourishing | SFI-12 | **Flourish** ^2^**:** VanderWeele proposed a 12-item secure flourishing Index (SFI) that measures wellbeing in each of the 6 domains and the overall wellbeing across these domains. All items were self-reported on a scale 0-10. Financial and material stability item wording was effectively reverse coded, such that a higher score indicates greater wellbeing. 1. How satisfied are you with your life as a whole these days? 2. How happy or unhappy do you usually feel? 3. How would you rate your physical health? 4. How would you rate your overall mental health? 5. To what extent do you feel the things you do in your life are worthwhile? 6. I understand my purpose in life. 7. I always act to promote good in all circumstances, even in difficult and challenging situations. 8. I am always able to give up some happiness now for greater happiness later. 9. I am content with my friendships and relationships. 10. My relationships are as satisfying as I would want them to be. 11. How frequently do you worry about being able to meet normal monthly living expenses? 12. How frequently do you worry about safety, food, or housing? Q1 (0 = Not Satisfied at All, 10 = Completely Satisfied); Q2 (0 = Extreme Unhappy, 10 = Extremely Happy); Q3 and Q4 (0=Poor,10=Excellent); Q5 (0=Not at All Worthwhile, 10=Completely Worthwhile); Q6, Q9, and Q10 (0 = Strongly Disagree, 10 = Strongly Agree); Q7 and Q8 (0 = Not True of Me, 10 = Completely True of Me); Q11 and Q12 (0 = Worry All of the Time, 10 = Do Not Ever. The measurement has good reliability in this work (α=0.65). |
|  | Optimism | Optimism | **Optimism** ^3,4^**:** To what extent do you agree with the following opinions? 1. You are always optimistic about your future 2. You want more good things to happen to you than bad things 3. You rarely expect good things to happen to you (reverse coded). The above questions use 5 points (0: strongly agree; 1: Agree; 2: It does not matter whether you agree or disagree; 3: Disagree; 4: Strongly disagree) Score range from 0 to 12, with higher scores indicating higher levels of optimism. The measurement has good reliability in this work (α= 0.692). |
|  | Mastery | Mastery | **Mastery** ^5^**:** Mastery was measured based on responses to 2 items: 1.Whether or not you’re able to get what you want is in yoour own hands;2.What happens to you in the future mostly depends on you. The above questions use 5 points (0: always; 1: Often; 2: Sometimes; 3: Very few; 4: never) score, by averaging the results of all items, the range is 0-8, the higher the score indicates the higher the control. The measurement has good reliability in this work (α=0.778). |
| Psychological distress | Depression | CESD-8 | **CES-D** ^6^**:** The scale was comprised of eight dichotomous items related to symptoms during the past week, including: felt depressed, everything was an effort, sleep was restless, was happy(reverse coded), felt lonely, felt sad, could not get going, and enjoyed life (reverse coded). The resulting CES-D score was a count of total number of symptoms (ranging from 0 to 8). The measurement has good reliability in this work (α= 0.786). |
|  | Anxiety | GAD-7 | **GAD** ^7^**:** The Generalized Anxiety Disorder 7-item (GAD-7) is a easy to perform initial screening tool for generalized anxiety disorder. Over the last 2 weeks, how often have you been bothered by the following problems? 1.Feeling nervous, anxious or on edge2.Not being able to stop or control worrying3.Worrying too much about different things. 4. Trouble relaxing. 5.Being so restless that it is hard to sit still. 6.Becoming easily annoyed or irritable. 7. Feeling afraid as if something awful might happen. The above questions use 5 points (0: Not at all; 1: Several days; 2: More than half the days; 3: More than half the days), and the results of all the items were summed to obtain a total score ranging from 0 to 21, with a higher score indicating anxiety. Cronbach coefficient α is 0.919, indicating good internal consistency. |
|  | Hopelessness | Hopelessness | **Hopelessness** ^8,9^**:** Hopelessness was measured with 2 questionnaire items from two previously validated scale. 1. The future seems hopelessness and you don't believe things are getting better. 2. It's no use trying to get what you want because you might not get it. The above questions use 5 points (4: always; 3: Often; 2: Sometimes; 1: Very few;0: Never), by averaging the results of all items, the range is 0-8. To match the other personal resource measures, the higher scores indicate high levels of hopelessness. The measurement has good reliability in this work (α= 0.792). |
| Social integrations | Social support | F-SozU K-6 | **F-SozU K-6** ^10^**:** Perceived social support plays a crucial role in the prevention of mental and physical diseases, and F-SozU-K-6 is a reliable, effective, and economical tool for assessing perceived social support, which can be effectively applied in clinical epidemiological studies or related fields. The questionnaire includes 1. I receive a lot of understanding and security from others. 2. There is someone very close to me whose help I can always count on. 3. If I need to, I can borrow something from friends or neighbors without any problems. 4. I know several people with whom I like to do things. 5. When I am sick, I can ask friends/relatives to handle important things for me without hesitation. 6. If I’m very depressed, I know who I can turn to. The above questions range from strongly disagree to strongly agree, and are expressed as 0-5 points. The total score is obtained by summing the results of all items, and the range is 0-30. The higher the score, the higher the level of social support. The scale showed good reliability, with an internal consistency of 0.896 for this sample. |
|  | Marital satisfaction | C-KMS-3 | **C-KMS** ^11^**:** Marital satisfaction is measured by three items: 1. How satisfied are you with your marriage, 2. How satisfied are you with your husband/wife as a spouse, and 3. how satisfied are you with your relationship? The items were scored on a scale of 0 (extremely dissatisfied) to 6 (extremely satisfied), and the results of all items were summed to produce an overall score on a scale of 0-18, with higher scores indicating higher marital satisfaction. The reliability and validity indexes were satisfactory (α= 0.83). |
| Another factor | Self-rated health | SRH | **SRH** ^12^**:** Self-rated health (SRH) refers to a person's subjective assessment of their own health status and is associated with a variety of outcomes such as morbidity and mortality. "How would you describe your health over the past year?" A 5-point scale from 0 (very good) to 4 (very poor) was used. SRH is coded in reverse, so a lower score indicates better health. |

| **Table B** Characteristics described by sex | | | | |
| --- | --- | --- | --- | --- |
|  | | **Sex** | |  |
| **Characteristic** | **Overall**  N = 538^1^ | **Female**  N = 329^1^ | **Male**  N = 209^1^ | **p-value**^2^ |
| **Social demographic:** |  |  |  |  |
| **Age** | 66.10 (12.04) | 67.14 (11.97) | 64.47 (12.00) | 0.029 |
| **Education attainment** |  |  |  | <0.001 |
| Illiteracy | 407 (76%) | 290 (88%) | 117 (56%) |  |
| Literacy | 131 (24%) | 39 (12%) | 92 (44%) |  |
| **Marital status** |  |  |  | <0.001 |
| Married | 466 (87%) | 272 (83%) | 194 (93%) |  |
| Unmarried | 11 (2.0%) | 7 (2.1%) | 4 (1.9%) |  |
| Widow/widower | 61 (11%) | 50 (15%) | 11 (5.3%) |  |
| **No. Yaks** | 36.25 (38.62) | 35.40 (38.69) | 37.81 (38.45) | 0.300 |
| **Annual expenditure** |  |  |  | 0.15 |
| <19999 | 169 (31%) | 105 (32%) | 64 (31%) |  |
| 20000-34999 | 216 (40%) | 140 (43%) | 76 (36%) |  |
| >35000 | 153 (28%) | 84 (26%) | 69 (33%) |  |
| **Pray time (min/day)** | 105.18 (87.89) | 110.78 (90.39) | 96.35 (83.24) | 0.038 |
| **Kowtow time (min/day)** | 22.26 (29.31) | 26.00 (30.21) | 16.36 (26.87) | <0.001 |
| **Pilgrimage time (day/year)** | 19.88 (47.79) | 18.32 (42.44) | 22.31 (55.14) | 0.3 |
| **Mental Health:** |  |  |  |  |
| **Psychological Well-Being** |  |  |  |  |
| SRS (range:0-22) | 15.74 (3.05) | 14.31 (2.16) | 15.20 (2.36) | <0.001 |
| Flourish (range:0-120) | 81.39 (11.37) | 80.87 (11.47) | 82.21 (11.19) | 0.2 |
| Optimism (range:0-12) | 7.71 (1.57) | 7.68 (1.44) | 7.76 (1.77) | 0.2 |
| Mastery (range:0-8) | 4.63 (1.47) | 4.30 (1.34) | 5.14 (1.52) | <0.001 |
| **Psychological Distress** |  |  |  |  |
| CESD (range:0-8) | 2.78 (2.18) | 2.90 (2.25) | 2.58 (2.06) | 0.100 |
| GAD (range:0-21) | 2.84 (3.85) | 2.98 (3.91) | 2.63 (3.76) | 0.130 |
| Hopeless (range:0-8) | 5.08 (1.45) | 5.12 (1.36) | 5.01 (1.57) | 0.800 |
| **Social relationships** |  |  |  |  |
| F_SozU (range:1-30) | 24.84 (4.14) | 24.87 (4.27) | 24.80 (3.94) | 0.600 |
| C_KMS (range:0-18) | 14.38 (1.60) | 14.29 (1.71) | 14.51 (1.41) | 0.400 |
| **Other factors** |  |  |  |  |
| SRH (range:0-4) | 2.52 (1.15) | 2.40 (1.19) | 2.70 (1.07) | 0.006 |
| **Physical activity and function:** |  |  |  |  |
| **BMIcal1** |  |  |  | 0.600 |
| < 25 | 256 (53%) | 155 (52%) | 101 (55%) |  |
| ≥25 | 227 (47%) | 143 (48%) | 84 (45%) |  |
| **SBP** |  |  |  | 0.008 |
| < 140 | 397 (74%) | 256 (78%) | 141 (68%) |  |
| ≥140 | 139 (26%) | 72 (22%) | 67 (32%) |  |
| **MVPA (h/day)** | 3.28 (1.91) | 3.34 (1.76) | 3.19 (2.12) | 0.200 |
| **Sleep duration (h/day)** | 7.84 (1.19) | 7.67 (1.22) | 8.12 (1.08) | <0.001 |
| **Grip strength (Kg)** | 20.79 (10.70) | 15.81 (6.63) | 28.63 (11.21) | <0.001 |
| **Gait speed (4m) (m/s)** | 0.86 (0.25) | 0.82 (0.24) | 0.94 (0.23) | <0.001 |
| **Endurance (2min) (m)** | 90.68 (23.91) | 84.21 (21.12) | 101.00 (24.52) | <0.001 |
| **Disease:** |  |  |  |  |
| **Arthritis** | 123 (23%) | 86 (26%) | 37 (18%) | 0.023 |
| **Heart disease** | 79 (15%) | 57 (17%) | 22 (11%) | 0.030 |
| **Chronic bronchitis** | 73 (14%) | 41 (12%) | 32 (15%) | 0.3 |
| **Insomnia** | 165 (31%) | 121 (37%) | 44 (21%) | <0.001 |
| **Health behavior:** |  |  |  |  |
| **Smoke Status** |  |  |  | <0.001 |
| Never | 469 (87%) | 327 (99%) | 142 (68%) |  |
| Past | 40 (7.4%) | 0 (0%) | 40 (19%) |  |
| Current | 29 (5.4%) | 2 (0.6%) | 27 (13%) |  |
|  |  |  |  |  |
| **Drink Status** |  |  |  | <0.001 |
| Never | 484 (90%) | 325 (99%) | 159 (76%) |  |
| Past | 42 (7.8%) | 3 (0.9%) | 39 (19%) |  |
| Current | 12 (2.2%) | 1 (0.3%) | 11 (5.1%) |  |
| ^1^Mean (SD); n (%) | | | | |
| ^2^Wilcoxon rank sum test; Pearson's Chi-squared test; Fisher's exact test | | | | |

**Table C** Multiple linear regression analyses of religious practices (praying, kowtow, and pilgrimage) on psychological resilience among older Amdo Tibetans.

|  | **Crude** | | **Multivariable adjusted** | | **Full adjusted** | |
| --- | --- | --- | --- | --- | --- | --- |
| *Predictors* | *Beta* | *SE* | *Beta* | *SE* | *Beta* | *SE* |
| (Intercept) | 15.52 ^***^ | 0.59 | 16.99 ^***^ | 1.04 | 16.33 ^***^ | 1.07 |
| pray orNot [yes] | 0.26 | 0.61 | 0.20 | 0.58 | 0.41 | 0.59 |
| kowtow orNot [yes] | 0.65 ^*^ | 0.28 | 0.85 ^**^ | 0.27 | 0.83 ^**^ | 0.28 |
| pilgrimage orNot [yes] | -0.84 ^**^ | 0.26 | -0.66 ^*^ | 0.26 | -0.64 ^*^ | 0.26 |
| age |  |  | -0.03 ^*^ | 0.01 | -0.02 | 0.01 |
| gender [male] |  |  | 0.78 ^**^ | 0.29 | 0.89 ^**^ | 0.32 |
| yak |  |  | -0.00 | 0.00 | -0.00 | 0.00 |
| expense_yearly120000-34999 |  |  | 0.95 ^**^ | 0.30 | 0.90 ^**^ | 0.30 |
| expense yearly1 [>35000] |  |  | 1.46 ^***^ | 0.33 | 1.44 ^***^ | 0.33 |
| siblings |  |  | -0.09 | 0.06 | -0.07 | 0.06 |
| marital status2  [unmarried] |  |  | -0.37 | 0.88 | -0.41 | 0.87 |
| marital status2  [widow/widower] |  |  | -0.53 | 0.40 | -0.56 | 0.40 |
| edu1 [literacy] |  |  | -0.63 | 0.33 | -0.65 ^*^ | 0.33 |
| householdsize |  |  | 0.02 | 0.05 | 0.03 | 0.05 |
| Distance to town |  |  | 0.00 | 0.01 | 0.00 | 0.01 |
| SBP1 [≥140] |  |  | -0.50 | 0.30 | -0.45 | 0.30 |
| BMIcal1 [≥25] |  |  | 0.03 | 0.25 | 0.02 | 0.25 |
| arthritis [yes] |  |  | -0.83 ^**^ | 0.30 | -0.85 ^**^ | 0.30 |
| heart [yes] |  |  | -0.68 | 0.38 | -0.69 | 0.37 |
| Chronic bronchitis [yes] |  |  | -0.42 | 0.37 | -0.36 | 0.37 |
| Insomnia [yes] |  |  | -1.00 ^***^ | 0.28 | -0.93 ^**^ | 0.29 |
| Smoke [Past] |  |  |  |  | -0.24 | 0.61 |
| Smoke [Current] |  |  |  |  | 0.98 | 0.61 |
| Drink [Past] |  |  |  |  | -0.93 | 0.58 |
| Drink [Current] |  |  |  |  | -0.09 | 0.89 |
| R^2^ / R^2^ adjusted | 0.025 / 0.020 | | 0.176 / 0.144 | | 0.188 / 0.150 | |
| ** p<0.05   ** p<0.01   *** p<0.001* | | | | | | |

| **Table D** Multiple linear regression analyses of religious practices (praying, kowtow, and pilgrimage) on flourishing (well-being) among older Amdo Tibetans. | | | | | | |
| --- | --- | --- | --- | --- | --- | --- |
|  | **Crude** | | **Multivariable adjusted** | | **Full adjusted** | |
| *Predictors* | *Beta* | *SE* | *Beta* | *SE* | *Beta* | *SE* |
| (Intercept) | 74.19 ^***^ | 2.21 | 70.86 ^***^ | 3.77 | 69.40 ^***^ | 3.90 |
| pray orNot [yes] | 4.73 ^*^ | 2.25 | 3.09 | 2.12 | 3.60 | 2.15 |
| kowtow orNot [yes] | 2.83 ^**^ | 1.04 | 3.22 ^**^ | 1.00 | 3.27 ^**^ | 1.00 |
| pilgrimage orNot [yes] | 1.65 ^*^ | 0.98 | 1.49 | 0.94 | 1.54 | 0.94 |
| age |  |  | 0.10 ^*^ | 0.04 | 0.11 ^*^ | 0.04 |
| gender [male] |  |  | 1.07 | 1.05 | 0.97 | 1.15 |
| yak |  |  | 0.00 | 0.01 | 0.00 | 0.01 |
| expense_yearly120000-34999 |  |  | 5.08 ^***^ | 1.09 | 5.03 ^***^ | 1.10 |
| expense yearly1 [>35000] |  |  | 5.12 ^***^ | 1.20 | 5.13 ^***^ | 1.21 |
| siblings |  |  | 0.29 | 0.22 | 0.32 | 0.23 |
| marital status2  [unmarried] |  |  | -3.98 | 3.18 | -4.20 | 3.19 |
| marital status2  [widow/widower] |  |  | -2.86 ^*^ | 1.46 | -2.87 ^*^ | 1.46 |
| edu1 [literacy] |  |  | -1.71 | 1.18 | -1.76 | 1.19 |
| householdsize |  |  | 0.21 | 0.19 | 0.21 | 0.19 |
| distanceHZ |  |  | -0.06 ^**^ | 0.02 | -0.06 ^**^ | 0.02 |
| SBP1 [≥140] |  |  | -0.71 | 1.09 | -0.55 | 1.10 |
| BMIcal1 [≥25] |  |  | -0.13 | 0.92 | -0.15 | 0.92 |
| arthritis [yes] |  |  | -5.33 ^***^ | 1.10 | -5.36 ^***^ | 1.10 |
| heart [yes] |  |  | -4.01 ^**^ | 1.36 | -4.03 ^**^ | 1.36 |
| chronic bronchitis [yes] |  |  | -0.04 | 1.32 | 0.16 | 1.33 |
| insomnia [yes] |  |  | -3.51 ^***^ | 1.03 | -3.44 ^**^ | 1.04 |
| smoke1 [Past] |  |  |  |  | 1.11 | 2.21 |
| smoke1 [Current] |  |  |  |  | 2.70 | 2.22 |
| drink1 [Past] |  |  |  |  | -2.48 | 2.11 |
| drink1 [Current] |  |  |  |  | 0.71 | 3.23 |
| R^2^ / R^2^ adjusted | 0.035 / 0.030 | | 0.219 / 0.189 | | 0.224 / 0.187 | |
| ** p<0.05   ** p<0.01   *** p<0.001* | | | | | | |

| **Table E** Multiple linear regression analyses of religious practices (praying, kowtow, and pilgrimage) on optimism among older Amdo Tibetans. | | | | | | |
| --- | --- | --- | --- | --- | --- | --- |
|  | **Crude** | | **Multivariable adjusted** | | **Full adjusted** | |
| *Predictors* | *Beta* | *SE* | *Beta* | *SE* | *Beta* | *SE* |
| (Intercept) | 6.76 ^***^ | 0.30 | 6.87 ^***^ | 0.56 | 7.01 ^***^ | 0.58 |
| pray orNot [yes] | 0.57 ^**^ | 0.31 | 0.39 | 0.31 | 0.34 | 0.32 |
| kowtow orNot [yes] | 0.56 ^***^ | 0.14 | 0.61 ^***^ | 0.15 | 0.61 ^***^ | 0.15 |
| pilgrimage orNot [yes] | 0.08 ^**^ | 0.14 | 0.15 | 0.14 | 0.15 | 0.14 |
| age |  |  | 0.00 | 0.01 | -0.00 | 0.01 |
| gender [male] |  |  | 0.27 | 0.15 | 0.27 | 0.17 |
| yak |  |  | -0.00 | 0.00 | -0.00 | 0.00 |
| expense_yearly120000-34999 |  |  | 0.57 ^***^ | 0.16 | 0.58 ^***^ | 0.16 |
| expense yearly1 [>35000] |  |  | 0.39 ^*^ | 0.18 | 0.40 ^*^ | 0.18 |
| siblings |  |  | -0.00 | 0.03 | -0.00 | 0.03 |
| marital status2  [unmarried] |  |  | -0.13 | 0.47 | -0.14 | 0.47 |
| marital status2  [widow/widower] |  |  | -0.35 | 0.22 | -0.34 | 0.22 |
| edu1 [literacy] |  |  | -0.47 ^**^ | 0.17 | -0.48 ^**^ | 0.18 |
| householdsize |  |  | 0.02 | 0.03 | 0.02 | 0.03 |
| distanceHZ |  |  | -0.00 | 0.00 | -0.00 | 0.00 |
| SBP1 [≥140] |  |  | -0.15 | 0.16 | -0.14 | 0.16 |
| BMIcal1 [≥25] |  |  | 0.01 | 0.14 | 0.01 | 0.14 |
| arthritis [yes] |  |  | -0.42 ^*^ | 0.16 | -0.43 ^**^ | 0.16 |
| heart [yes] |  |  | -0.01 | 0.20 | -0.02 | 0.20 |
| chronic bronchitis [yes] |  |  | 0.24 | 0.20 | 0.24 | 0.20 |
| insomnia [yes] |  |  | -0.16 | 0.15 | -0.17 | 0.15 |
| smoke1 [Past] |  |  |  |  | -0.10 | 0.33 |
| smoke1 [Current] |  |  |  |  | -0.26 | 0.33 |
| drink1 [Past] |  |  |  |  | 0.12 | 0.31 |
| drink1 [Current] |  |  |  |  | 0.48 | 0.48 |
| R^2^ / R^2^ adjusted | 0.042 / 0.037 | | 0.111 / 0.077 | | 0.114 / 0.072 | |
| ** p<0.05   ** p<0.01   *** p<0.001* | | | | | | |

| **Table F** Multiple linear regression analyses of religious practices (praying, kowtow, and pilgrimage) on mastery among older Amdo Tibetans. | | | | | | |
| --- | --- | --- | --- | --- | --- | --- |
|  | **Crude** | | **Multivariable adjusted** | | **Full adjusted** | |
| *Predictors* | *Beta* | *SE* | *Beta* | *SE* | *Beta* | *SE* |
| (Intercept) | 4.97 ^***^ | 0.29 | 4.37 ^***^ | 0.51 | 4.42 ^***^ | 0.52 |
| pray orNot [yes] | -0.40 | 0.29 | -0.39 | 0.29 | -0.36 | 0.29 |
| kowtow orNot [yes] | 0.19 ^*^ | 0.14 | 0.33 ^*^ | 0.13 | 0.37 ^**^ | 0.13 |
| pilgrimage orNot [yes] | -0.16 | 0.13 | -0.23 | 0.13 | -0.24 | 0.13 |
| age |  |  | 0.00 | 0.01 | 0.00 | 0.01 |
| gender [male] |  |  | 0.75 ^***^ | 0.14 | 0.60 ^***^ | 0.15 |
| yak |  |  | -0.00 | 0.00 | -0.00 | 0.00 |
| expense_yearly120000-34999 |  |  | 0.50 ^***^ | 0.15 | 0.53 ^***^ | 0.15 |
| expense yearly1 [>35000] |  |  | 0.55 ^***^ | 0.16 | 0.54 ^***^ | 0.16 |
| siblings |  |  | 0.05 | 0.03 | 0.03 | 0.03 |
| marital status2  [unmarried] |  |  | -0.06 | 0.43 | -0.03 | 0.43 |
| marital status2  [widow/widower] |  |  | 0.09 | 0.20 | 0.09 | 0.19 |
| edu1 [literacy] |  |  | 0.15 | 0.16 | 0.19 | 0.16 |
| householdsize |  |  | 0.00 | 0.03 | -0.01 | 0.03 |
| distanceHZ |  |  | -0.00 | 0.00 | -0.00 | 0.00 |
| SBP1 [≥140] |  |  | -0.09 | 0.15 | -0.14 | 0.15 |
| BMIcal1 [≥25] |  |  | 0.11 | 0.12 | 0.12 | 0.12 |
| arthritis [yes] |  |  | -0.09 | 0.15 | -0.05 | 0.15 |
| heart [yes] |  |  | -0.38 ^*^ | 0.18 | -0.36 | 0.18 |
| chronic bronchitis [yes] |  |  | 0.25 | 0.18 | 0.21 | 0.18 |
| insomnia [yes] |  |  | -0.22 | 0.14 | -0.26 | 0.14 |
| smoke1 [Past] |  |  |  |  | 0.67 ^*^ | 0.30 |
| smoke1 [Current] |  |  |  |  | 0.26 | 0.30 |
| drink1 [Past] |  |  |  |  | 0.32 | 0.28 |
| drink1 [Current] |  |  |  |  | -1.06 ^*^ | 0.43 |
| R^2^ / R^2^ adjusted | 0.008 / 0.003 | | 0.149 / 0.116 | | 0.177 / 0.139 | |
| ** p<0.05   ** p<0.01   *** p<0.001* | | | | | | |

| **Table G** Multiple linear regression analyses of religious practices (praying, kowtow, and pilgrimage) on depression among older Amdo Tibetans. | | | | | | |
| --- | --- | --- | --- | --- | --- | --- |
|  | **Crude** | | **Multivariable adjusted** | | **Full adjusted** | |
| *Predictors* | *Beta* | *SE* | *Beta* | *SE* | *Beta* | *SE* |
| (Intercept) | 2.92 ^***^ | 0.43 | 1.28 | 0.70 | 1.58 ^*^ | 0.72 |
| pray orNot [yes] | 0.24 | 0.44 | 0.32 | 0.39 | 0.25 | 0.40 |
| kowtow orNot [yes] | -0.51 ^*^ | 0.20 | -0.46 ^*^ | 0.18 | -0.44 ^*^ | 0.18 |
| pilgrimage orNot [yes] | -0.08 | 0.19 | -0.16 | 0.17 | -0.18 | 0.17 |
| age |  |  | 0.02 ^*^ | 0.01 | 0.01 | 0.01 |
| gender [male] |  |  | 0.06 | 0.19 | -0.05 | 0.21 |
| yak |  |  | 0.00 | 0.00 | 0.00 | 0.00 |
| expense_yearly120000-34999 |  |  | -0.69 ^***^ | 0.20 | -0.67 ^**^ | 0.20 |
| expense yearly1 [>35000] |  |  | -0.54 ^*^ | 0.22 | -0.54 ^*^ | 0.22 |
| siblings |  |  | 0.07 | 0.04 | 0.05 | 0.04 |
| marital status2  [unmarried] |  |  | -0.77 | 0.59 | -0.75 | 0.59 |
| marital status2  [widow/widower] |  |  | 0.40 | 0.27 | 0.40 | 0.27 |
| edu1 [literacy] |  |  | -0.00 | 0.22 | 0.02 | 0.22 |
| householdsize |  |  | -0.04 | 0.03 | -0.04 | 0.03 |
| distanceHZ |  |  | 0.00 | 0.00 | 0.00 | 0.00 |
| SBP1 [≥140] |  |  | 0.04 | 0.20 | 0.00 | 0.20 |
| BMIcal1 [≥25] |  |  | -0.08 | 0.17 | -0.07 | 0.17 |
| arthritis [yes] |  |  | 0.65 ^**^ | 0.20 | 0.67 ^**^ | 0.20 |
| heart [yes] |  |  | 0.71 ^**^ | 0.25 | 0.73 ^**^ | 0.25 |
| chronic bronchitis [yes] |  |  | -0.38 | 0.25 | -0.43 | 0.25 |
| insomnia [yes] |  |  | 1.79 ^***^ | 0.19 | 1.75 ^***^ | 0.19 |
| smoke1 [Past] |  |  |  |  | 0.23 | 0.41 |
| smoke1 [Current] |  |  |  |  | -0.28 | 0.41 |
| drink1 [Past] |  |  |  |  | 0.65 | 0.39 |
| drink1 [Current] |  |  |  |  | -0.29 | 0.60 |
| R^2^ / R^2^ adjusted | 0.013 / 0.007 | | 0.272 / 0.244 | | 0.281 / 0.248 | |
| ** p<0.05   ** p<0.01   *** p<0.001* | | | | | | |

| **Table H** Multiple linear regression analyses of religious practices (praying, kowtow, and pilgrimage) on anxiety among older Amdo Tibetans. | | | | | | |
| --- | --- | --- | --- | --- | --- | --- |
|  | **Crude** | | **Multivariable adjusted** | | **Full adjusted** | |
| *Predictors* | *Beta* | *SE* | *Beta* | *SE* | *Beta* | *SE* |
| (Intercept) | 5.22 ^***^ | 0.75 | 2.73 ^*^ | 1.29 | 3.34 ^*^ | 1.33 |
| pray orNot [yes] | -2.53 ^**^ | 0.77 | -2.20 ^**^ | 0.73 | -2.33 ^**^ | 0.73 |
| kowtow orNot [yes] | -0.08 | 0.35 | -0.24 | 0.34 | -0.22 | 0.34 |
| pilgrimage orNot [yes] | 0.15 | 0.33 | -0.00 | 0.32 | -0.06 | 0.32 |
| age |  |  | 0.00 | 0.02 | -0.00 | 0.02 |
| gender [male] |  |  | -0.38 | 0.36 | -0.55 | 0.39 |
| yak |  |  | 0.01 | 0.00 | 0.01 | 0.00 |
| expense_yearly120000-34999 |  |  | -1.71 ^***^ | 0.37 | -1.68 ^***^ | 0.37 |
| expense yearly1 [>35000] |  |  | -2.20 ^***^ | 0.41 | -2.22 ^***^ | 0.41 |
| siblings |  |  | 0.09 | 0.08 | 0.04 | 0.08 |
| marital status2  [unmarried] |  |  | -0.51 | 1.09 | -0.57 | 1.09 |
| marital status2  [widow/widower] |  |  | 0.29 | 0.50 | 0.28 | 0.50 |
| edu1 [literacy] |  |  | 0.70 | 0.41 | 0.66 | 0.41 |
| householdsize |  |  | 0.03 | 0.06 | 0.03 | 0.06 |
| distanceHZ |  |  | 0.02 ^**^ | 0.01 | 0.02 ^**^ | 0.01 |
| SBP1 [≥140] |  |  | 0.59 | 0.37 | 0.56 | 0.38 |
| BMIcal1 [≥25] |  |  | -0.12 | 0.32 | -0.08 | 0.32 |
| arthritis [yes] |  |  | 0.76 ^*^ | 0.38 | 0.77 ^*^ | 0.37 |
| heart [yes] |  |  | 1.29 ^**^ | 0.47 | 1.33 ^**^ | 0.47 |
| chronic bronchitis [yes] |  |  | 1.44 ^**^ | 0.45 | 1.37 ^**^ | 0.45 |
| insomnia [yes] |  |  | 1.40 ^***^ | 0.35 | 1.37 ^***^ | 0.35 |
| smoke1 [Past] |  |  |  |  | -1.03 | 0.75 |
| smoke1 [Current] |  |  |  |  | -0.44 | 0.76 |
| drink1 [Past] |  |  |  |  | 2.16 ^**^ | 0.72 |
| drink1 [Current] |  |  |  |  | 1.15 | 1.10 |
| R^2^ / R^2^ adjusted | 0.021 / 0.016 | | 0.199 / 0.168 | | 0.213 / 0.176 | |
| ** p<0.05   ** p<0.01   *** p<0.001* | | | | | | |

| **Table I** Multiple linear regression analyses of religious practices (praying, kowtow, and pilgrimage) on hopelessness among older Amdo Tibetans. | | | | | | |
| --- | --- | --- | --- | --- | --- | --- |
|  | **Crude** | | **Multivariable adjusted** | | **Full adjusted** | |
| *Predictors* | *Beta* | *SE* | *Beta* | *SE* | *Beta* | *SE* |
| (Intercept) | 5.00 ^***^ | 0.28 | 4.53 ^***^ | 0.52 | 4.58 ^***^ | 0.53 |
| pray orNot [yes] | 0.49 | 0.29 | 0.41 | 0.29 | 0.40 | 0.29 |
| kowtow orNot [yes] | -0.40 ^**^ | 0.13 | -0.34 ^*^ | 0.14 | -0.33 ^*^ | 0.14 |
| pilgrimage orNot [yes] | -0.25 ^*^ | 0.13 | -0.37 ^**^ | 0.13 | -0.37 ^**^ | 0.13 |
| age |  |  | 0.01 ^*^ | 0.01 | 0.01 ^*^ | 0.01 |
| gender [male] |  |  | -0.12 | 0.14 | -0.15 | 0.16 |
| yak |  |  | -0.00 | 0.00 | -0.00 | 0.00 |
| expense_yearly120000-34999 |  |  | -0.12 | 0.15 | -0.11 | 0.15 |
| expense yearly1 [>35000] |  |  | 0.24 | 0.16 | 0.24 | 0.16 |
| siblings |  |  | 0.10 ^**^ | 0.03 | 0.10 ^**^ | 0.03 |
| marital status2  [unmarried] |  |  | -0.46 | 0.43 | -0.41 | 0.43 |
| marital status2  [widow/widower] |  |  | 0.07 | 0.20 | 0.07 | 0.20 |
| householdsize |  |  | -0.00 | 0.03 | -0.01 | 0.03 |
| edu1 [literacy] |  |  | -0.10 | 0.16 | -0.07 | 0.16 |
| distanceHZ |  |  | -0.00 | 0.00 | -0.00 | 0.00 |
| SBP1 [≥140] |  |  | 0.12 | 0.15 | 0.09 | 0.15 |
| BMIcal1 [≥25] |  |  | -0.03 | 0.13 | -0.03 | 0.13 |
| arthritis [yes] |  |  | 0.27 | 0.15 | 0.29 | 0.15 |
| heart [yes] |  |  | -0.09 | 0.19 | -0.09 | 0.19 |
| chronic bronchitis [yes] |  |  | -0.37 ^*^ | 0.18 | -0.40 ^*^ | 0.18 |
| insomnia [yes] |  |  | -0.01 | 0.14 | -0.04 | 0.14 |
| smoke1 [Past] |  |  |  |  | 0.45 | 0.30 |
| smoke1 [Current] |  |  |  |  | -0.05 | 0.30 |
| drink1 [Past] |  |  |  |  | -0.05 | 0.29 |
| drink1 [Current] |  |  |  |  | -0.86 | 0.44 |
| R^2^ / R^2^ adjusted | 0.030 / 0.024 | | 0.095 / 0.061 | | 0.107 / 0.065 | |
| ** p<0.05   ** p<0.01   *** p<0.001* | | | | | | |

| **Table J** Multiple linear regression analyses of religious practices (praying, kowtow, and pilgrimage) on social support among older Amdo Tibetans. | | | | | | |
| --- | --- | --- | --- | --- | --- | --- |
|  | **Crude** | | **Multivariable adjusted** | | **Full adjusted** | |
| *Predictors* | *Beta* | *SE* | *Beta* | *SE* | *Beta* | *SE* |
| (Intercept) | 22.68 ^***^ | 0.81 | 24.93 ^***^ | 1.43 | 23.60 ^***^ | 1.46 |
| pray orNot [yes] | 2.60 ^**^ | 0.82 | 1.74 ^*^ | 0.80 | 2.16 ^**^ | 0.80 |
| kowtow orNot [yes] | -0.01 | 0.38 | 0.16 | 0.38 | 0.14 | 0.37 |
| pilgrimage orNot [yes] | -0.57 | 0.36 | -0.21 | 0.36 | -0.17 | 0.35 |
| age |  |  | -0.02 | 0.02 | -0.01 | 0.02 |
| gender [male] |  |  | -0.21 | 0.40 | -0.06 | 0.43 |
| yak |  |  | -0.00 | 0.00 | -0.00 | 0.00 |
| expense_yearly120000-34999 |  |  | 1.08 ^**^ | 0.41 | 1.00 ^*^ | 0.41 |
| expense yearly1 [>35000] |  |  | 1.93 ^***^ | 0.46 | 1.92 ^***^ | 0.45 |
| siblings |  |  | -0.13 | 0.08 | -0.09 | 0.08 |
| marital status2  [unmarried] |  |  | 1.30 | 1.20 | 1.11 | 1.19 |
| marital status2  [widow/widower] |  |  | -0.14 | 0.55 | -0.16 | 0.54 |
| edu1 [literacy] |  |  | -0.51 | 0.45 | -0.60 | 0.44 |
| householdsize |  |  | 0.01 | 0.07 | 0.02 | 0.07 |
| distanceHZ |  |  | -0.00 | 0.01 | -0.00 | 0.01 |
| SBP1 [≥140] |  |  | 0.13 | 0.41 | 0.29 | 0.41 |
| BMIcal1 [≥25] |  |  | 0.00 | 0.35 | -0.01 | 0.34 |
| arthritis [yes] |  |  | -2.73 ^***^ | 0.42 | -2.78 ^***^ | 0.41 |
| heart [yes] |  |  | 0.42 | 0.52 | 0.38 | 0.51 |
| chronic bronchitis [yes] |  |  | 1.18 ^*^ | 0.50 | 1.35 ^**^ | 0.50 |
| insomnia [yes] |  |  | -0.57 | 0.39 | -0.43 | 0.39 |
| smoke1 [Past] |  |  |  |  | -0.40 | 0.82 |
| smoke1 [Current] |  |  |  |  | 2.08 ^*^ | 0.83 |
| drink1 [Past] |  |  |  |  | -1.98 ^*^ | 0.79 |
| drink1 [Current] |  |  |  |  | 1.17 | 1.21 |
| R^2^ / R^2^ adjusted | 0.022 / 0.017 | | 0.153 / 0.120 | | 0.186 / 0.147 | |
| ** p<0.05   ** p<0.01   *** p<0.001* | | | | | | |

| **Table K** Multiple linear regression analyses of religious practices (praying, kowtow, and pilgrimage) on marital satisfaction among older Amdo Tibetans. | | | | | | |
| --- | --- | --- | --- | --- | --- | --- |
|  | **Crude** | | **Multivariable adjusted** | | **Full adjusted** | |
| *Predictors* | *Beta* | *SE* | *Beta* | *SE* | *Beta* | *SE* |
| (Intercept) | 13.88 ^***^ | 0.32 | 15.07 ^***^ | 0.56 | 14.73 ^***^ | 0.58 |
| pray orNot [yes] | 0.78 ^*^ | 0.33 | 0.57 | 0.32 | 0.70 ^*^ | 0.32 |
| kowtow orNot [yes] | -0.28 | 0.15 | -0.19 | 0.15 | -0.18 | 0.15 |
| pilgrimage orNot [yes] | -0.14 | 0.14 | -0.12 | 0.14 | -0.11 | 0.14 |
| age |  |  | -0.00 | 0.01 | 0.00 | 0.01 |
| gender [male] |  |  | 0.08 | 0.16 | 0.04 | 0.17 |
| yak |  |  | 0.00 | 0.00 | 0.00 | 0.00 |
| expense_yearly120000-34999 |  |  | 0.26 | 0.16 | 0.25 | 0.16 |
| expense yearly1 [>35000] |  |  | 0.60 ^***^ | 0.18 | 0.59 ^**^ | 0.18 |
| siblings |  |  | 0.01 | 0.03 | 0.01 | 0.03 |
| marital status2  [unmarried] |  |  | -0.04 | 0.48 | -0.11 | 0.48 |
| marital status2  [widow/widower] |  |  | -0.45 ^*^ | 0.22 | -0.46 ^*^ | 0.22 |
| edu1 [literacy] |  |  | -0.14 | 0.18 | -0.16 | 0.18 |
| householdsize |  |  | 0.03 | 0.03 | 0.03 | 0.03 |
| distanceHZ |  |  | -0.02 ^***^ | 0.00 | -0.02 ^***^ | 0.00 |
| SBP1 [≥140] |  |  | 0.29 | 0.16 | 0.32 | 0.16 |
| BMIcal1 [≥25] |  |  | -0.24 | 0.14 | -0.24 | 0.14 |
| arthritis [yes] |  |  | -0.42 ^*^ | 0.16 | -0.43 ^**^ | 0.16 |
| heart [yes] |  |  | 0.12 | 0.20 | 0.12 | 0.20 |
| chronic bronchitis [yes] |  |  | -0.08 | 0.20 | -0.04 | 0.20 |
| insomnia [yes] |  |  | -0.24 | 0.15 | -0.22 | 0.16 |
| smoke1 [Past] |  |  |  |  | 0.06 | 0.33 |
| smoke1 [Current] |  |  |  |  | 0.74 ^*^ | 0.33 |
| drink1 [Past] |  |  |  |  | -0.27 | 0.31 |
| drink1 [Current] |  |  |  |  | 0.21 | 0.48 |
| R^2^ / R^2^ adjusted | 0.017 / 0.011 | | 0.164 / 0.132 | | 0.175 / 0.137 | |
| ** p<0.05   ** p<0.01   *** p<0.001* | | | | | | |

| **Table L** Multiple linear regression analyses of religious practices (praying, kowtow, and pilgrimage) on self-related health among older Amdo Tibetans. | | | | | | |
| --- | --- | --- | --- | --- | --- | --- |
|  | **Crude** | | **Multivariable adjusted** | | **Full adjusted** | |
| *Predictors* | *Beta* | *SE* | *Beta* | *SE* | *Beta* | *SE* |
| (Intercept) | 2.64 ^***^ | 0.22 | 2.71 ^***^ | 0.40 | 2.60 ^***^ | 0.41 |
| pray orNot [yes] | -0.45 | 0.23 | -0.47 | 0.22 | -0.41 | 0.23 |
| kowtow orNot [yes] | 0.48 ^***^ | 0.11 | 0.53 ^***^ | 0.10 | 0.54 ^***^ | 0.10 |
| pilgrimage orNot [yes] | -0.02 | 0.10 | -0.06 | 0.10 | -0.07 | 0.10 |
| age |  |  | 0.01 | 0.00 | 0.01 | 0.00 |
| gender [male] |  |  | 0.28 ^*^ | 0.11 | 0.22 | 0.12 |
| yak |  |  | 0.00 | 0.00 | 0.00 | 0.00 |
| expense_yearly120000-34999 |  |  | 0.17 | 0.11 | 0.17 | 0.11 |
| expense yearly1 [>35000] |  |  | 0.08 | 0.13 | 0.08 | 0.13 |
| siblings |  |  | 0.02 | 0.02 | 0.02 | 0.02 |
| marital status2  [unmarried] |  |  | -0.39 | 0.33 | -0.43 | 0.33 |
| marital status2  [widow/widower] |  |  | -0.16 | 0.15 | -0.16 | 0.15 |
| edu1 [literacy] |  |  | -0.11 | 0.12 | -0.12 | 0.12 |
| householdsize |  |  | -0.00 | 0.02 | -0.00 | 0.02 |
| distanceHZ |  |  | -0.01 ^**^ | 0.00 | -0.01 ^**^ | 0.00 |
| SBP1 [≥140] |  |  | -0.03 | 0.11 | -0.02 | 0.12 |
| BMIcal1 [≥25] |  |  | -0.05 | 0.10 | -0.05 | 0.10 |
| arthritis [yes] |  |  | -0.29 ^*^ | 0.11 | -0.29 ^*^ | 0.12 |
| heart [yes] |  |  | -0.45 ^**^ | 0.14 | -0.45 ^**^ | 0.14 |
| chronic bronchitis [yes] |  |  | 0.27 ^*^ | 0.14 | 0.29 ^*^ | 0.14 |
| insomnia [yes] |  |  | -0.40 ^***^ | 0.11 | -0.39 ^***^ | 0.11 |
| smoke1 [Past] |  |  |  |  | 0.02 | 0.23 |
| smoke1 [Current] |  |  |  |  | 0.37 | 0.23 |
| drink1 [Past] |  |  |  |  | 0.05 | 0.22 |
| drink1 [Current] |  |  |  |  | 0.24 | 0.34 |
| R^2^ / R^2^ adjusted | 0.041 / 0.035 | | 0.169 / 0.137 | | 0.175 / 0.136 | |
| ** p<0.05   ** p<0.01   *** p<0.001* | | | | | | |

| **Table M** Multiple linear regression analyses of religious practices (praying, kowtow, and pilgrimage) on moderate to vigorous physical activity (h/day) among older Amdo Tibetans. | | | | | | |
| --- | --- | --- | --- | --- | --- | --- |
|  | **Crude** | | **Multivariable adjusted** | | **Full adjusted** | |
| *Predictors* | *Beta* | *SE* | *Beta* | *SE* | *Beta* | *SE* |
| (Intercept) | 4.93 ^***^ | 0.50 | 8.02 ^***^ | 0.94 | 8.19 ^***^ | 0.98 |
| pray orNot [yes] | -1.83 ^***^ | 0.52 | -1.35 ^**^ | 0.52 | -1.36 ^*^ | 0.52 |
| kowtow orNot [yes] | 0.59 ^*^ | 0.23 | 0.42 ^*^ | 0.23 | 0.44 ^*^ | 0.23 |
| pilgrimage orNot [yes] | -0.54 ^*^ | 0.22 | -0.51 ^*^ | 0.22 | -0.52 ^*^ | 0.22 |
| age |  |  | -0.04 ^***^ | 0.01 | -0.04 ^***^ | 0.01 |
| gender [male] |  |  | -0.32 | 0.25 | -0.38 | 0.27 |
| yak |  |  | -0.01 ^*^ | 0.00 | -0.01 ^*^ | 0.00 |
| expense yearly1 [>35000] |  |  | 0.64 ^*^ | 0.27 | 0.59 ^*^ | 0.28 |
| expense_yearly120000-34999 |  |  | 0.18 | 0.26 | 0.16 | 0.26 |
| siblings |  |  | -0.06 | 0.06 | -0.08 | 0.06 |
| marital status2  [unmarried] |  |  | 1.72 ^*^ | 0.82 | 1.93 ^*^ | 0.84 |
| marital status2  [widow_widower] |  |  | 0.43 | 0.39 | 0.44 | 0.39 |
| edu1 [literacy] |  |  | 0.06 | 0.28 | 0.12 | 0.28 |
| householdsize |  |  | -0.02 | 0.04 | -0.02 | 0.04 |
| distanceHZ |  |  | -0.00 | 0.01 | -0.00 | 0.01 |
| SBP1 [â‰¥140] |  |  | -0.12 | 0.27 | -0.19 | 0.28 |
| BMIcal1 [â‰¥25] |  |  | 0.16 | 0.21 | 0.16 | 0.22 |
| arthritis [yes] |  |  | 0.18 | 0.27 | 0.22 | 0.27 |
| heart [yes] |  |  | -0.60 ^*^ | 0.31 | -0.56 ^*^ | 0.31 |
| chronic bronchitis [yes] |  |  | -0.29 | 0.34 | -0.38 | 0.34 |
| insomnia [yes] |  |  | -0.45 ^*^ | 0.25 | -0.53 ^*^ | 0.25 |
| smoke1 [Past] |  |  |  |  | -0.04 | 0.50 |
| smoke1 [Current] |  |  |  |  | -0.16 | 0.46 |
| drink1 [Past] |  |  |  |  | 0.69 | 0.52 |
| drink1 [Current] |  |  |  |  | -1.39 | 0.97 |
| R^2^ / R^2^ adjusted | 0.068 / 0.059 | | 0.206 / 0.147 | | 0.223 / 0.152 | |
| ** p<0.05   ** p<0.01   *** p<0.001* | | | | | | |

| **Table N** Multiple linear regression analyses of religious practices (praying, kowtow, and pilgrimage) on sleep duration (h/day) among older Amdo Tibetans. | | | | | | |
| --- | --- | --- | --- | --- | --- | --- |
|  | **Crude** | | **Multivariable adjusted** | | **Full adjusted** | |
| *Predictors* | *Beta* | *SE* | *Beta* | *SE* | *Beta* | *SE* |
| (Intercept) | 7.60 ^***^ | 0.23 | 6.22 ^***^ | 0.42 | 6.14 ^***^ | 0.44 |
| pray orNot [yes] | -0.01 | 0.24 | -0.01 | 0.24 | 0.02 | 0.24 |
| kowtow orNot [yes] | 0.24 ^*^ | 0.11 | 0.34 ^**^ | 0.11 | 0.32 ^**^ | 0.11 |
| pilgrimage orNot [yes] | 0.15 | 0.10 | 0.01 | 0.10 | 0.01 | 0.10 |
| age |  |  | 0.02 ^***^ | 0.00 | 0.02 ^***^ | 0.01 |
| gender [male] |  |  | 0.49 ^***^ | 0.12 | 0.54 ^***^ | 0.13 |
| yak |  |  | 0.00 | 0.00 | 0.00 | 0.00 |
| expense_yearly120000-34999 |  |  | 0.04 | 0.12 | 0.03 | 0.12 |
| expense yearly1 [>35000] |  |  | -0.04 | 0.13 | -0.07 | 0.13 |
| siblings |  |  | 0.03 | 0.02 | 0.03 | 0.03 |
| marital status2  [unmarried] |  |  | 0.47 | 0.36 | 0.50 | 0.36 |
| marital status2  [widow/widower] |  |  | 0.18 | 0.16 | 0.16 | 0.16 |
| edu1 [literacy] |  |  | 0.05 | 0.13 | 0.06 | 0.13 |
| householdsize |  |  | 0.03 | 0.02 | 0.03 | 0.02 |
| distanceHZ |  |  | -0.00 | 0.00 | -0.00 | 0.00 |
| SBP1 [≥140] |  |  | -0.04 | 0.12 | -0.06 | 0.12 |
| BMIcal1 [≥25] |  |  | 0.10 | 0.10 | 0.11 | 0.10 |
| arthritis [yes] |  |  | -0.17 | 0.12 | -0.16 | 0.12 |
| heart [yes] |  |  | -0.15 | 0.15 | -0.15 | 0.15 |
| chronic bronchitis [yes] |  |  | 0.15 | 0.15 | 0.13 | 0.15 |
| insomnia [yes] |  |  | -0.22 | 0.12 | -0.20 | 0.12 |
| smoke1 [Past] |  |  |  |  | -0.40 | 0.25 |
| smoke1 [Current] |  |  |  |  | 0.09 | 0.25 |
| drink1 [Past] |  |  |  |  | 0.20 | 0.24 |
| drink1 [Current] |  |  |  |  | -0.49 | 0.36 |
| R^2^ / R^2^ adjusted | 0.015 / 0.010 | | 0.114 / 0.080 | | 0.124 / 0.083 | |
| ** p<0.05   ** p<0.01   *** p<0.001* | | | | | | |

| **Table O** Multiple linear regression analyses of religious practices (praying, kowtow, and pilgrimage) on grip strength (kg) among older Amdo Tibetans. | | | | | | |
| --- | --- | --- | --- | --- | --- | --- |
|  | **Crude** | | **Multivariable adjusted** | | **Full adjusted** | |
| *Predictors* | *Beta* | *SE* | *Beta* | *SE* | *Beta* | *SE* |
| (Intercept) | 28.36 ^***^ | 2.08 | 41.98 ^***^ | 2.67 | 40.82 ^***^ | 2.72 |
| pray orNot [yes] | -7.73 ^***^ | 2.12 | -3.88 ^**^ | 1.50 | -3.09 ^*^ | 1.50 |
| kowtow orNot [yes] | -0.22 | 0.98 | 1.64 ^*^ | 0.70 | 1.87 ^**^ | 0.70 |
| pilgrimage orNot [yes] | -0.49 | 0.93 | -0.37 | 0.66 | -0.47 | 0.65 |
| age |  |  | -0.31 ^***^ | 0.03 | -0.30 ^***^ | 0.03 |
| gender [male] |  |  | 11.47 ^***^ | 0.74 | 10.34 ^***^ | 0.80 |
| yak |  |  | -0.00 | 0.01 | -0.00 | 0.01 |
| expense_yearly120000-34999 |  |  | 0.97 | 0.77 | 1.01 | 0.76 |
| expense yearly1 [>35000] |  |  | 1.22 | 0.85 | 1.16 | 0.84 |
| siblings |  |  | 0.33 ^*^ | 0.16 | 0.20 | 0.16 |
| marital status2  [unmarried] |  |  | -6.04 ^**^ | 2.25 | -6.74 ^**^ | 2.22 |
| marital status2  [widow/widower] |  |  | 0.10 | 1.03 | 0.03 | 1.02 |
| edu1 [literacy] |  |  | 0.55 | 0.84 | 0.35 | 0.83 |
| householdsize |  |  | -0.06 | 0.13 | -0.07 | 0.13 |
| distanceHZ |  |  | -0.05 ^***^ | 0.02 | -0.06 ^***^ | 0.01 |
| SBP1 [≥140] |  |  | 0.15 | 0.77 | 0.33 | 0.77 |
| BMIcal1 [≥25] |  |  | 1.17 | 0.65 | 1.28 ^*^ | 0.64 |
| arthritis [yes] |  |  | -0.16 | 0.78 | -0.12 | 0.77 |
| heart [yes] |  |  | -3.76 ^***^ | 0.96 | -3.59 ^***^ | 0.95 |
| chronic bronchitis [yes] |  |  | -1.56 | 0.94 | -1.42 | 0.93 |
| insomnia [yes] |  |  | 1.12 | 0.73 | 1.14 | 0.72 |
| smoke1 [Past] |  |  |  |  | -0.01 | 1.54 |
| smoke1 [Current] |  |  |  |  | 5.39 ^***^ | 1.54 |
| drink1 [Past] |  |  |  |  | 2.72 | 1.47 |
| drink1 [Current] |  |  |  |  | 3.81 | 2.25 |
| R^2^ / R^2^ adjusted | 0.027 / 0.021 | | 0.556 / 0.538 | | 0.574 / 0.554 | |
| ** p<0.05   ** p<0.01   *** p<0.001* | | | | | | |

| **Table P** Multiple linear regression analyses of religious practices (praying, kowtow, and pilgrimage) on gait speed (m/s) among older Amdo Tibetans. | | | | | | |
| --- | --- | --- | --- | --- | --- | --- |
|  | **Crude** | | **Multivariable adjusted** | | **Full adjusted** | |
| *Predictors* | *Beta* | *SE* | *Beta* | *SE* | *Beta* | *SE* |
| (Intercept) | 0.95 ^***^ | 0.05 | 1.40 ^***^ | 0.09 | 1.43 ^***^ | 0.09 |
| pray orNot [yes] | -0.16 ^**^ | 0.05 | -0.06 | 0.05 | -0.07 | 0.05 |
| kowtow orNot [yes] | 0.06 ^*^ | 0.03 | 0.07 ^**^ | 0.02 | 0.08 ^***^ | 0.02 |
| pilgrimage orNot [yes] | 0.00 | 0.02 | 0.02 | 0.02 | 0.02 | 0.02 |
| age |  |  | -0.01 ^***^ | 0.00 | -0.01 ^***^ | 0.00 |
| gender [male] |  |  | 0.11 ^***^ | 0.02 | 0.10 ^***^ | 0.03 |
| yak |  |  | 0.00 | 0.00 | -0.00 | 0.00 |
| expense_yearly120000-34999 |  |  | -0.10 ^***^ | 0.03 | -0.10 ^***^ | 0.03 |
| expense yearly1 [>35000] |  |  | -0.07 ^*^ | 0.03 | -0.07 ^*^ | 0.03 |
| siblings |  |  | 0.01 | 0.01 | 0.00 | 0.01 |
| marital status2  [unmarried] |  |  | -0.13 | 0.07 | -0.13 | 0.07 |
| marital status2  [widow/widower] |  |  | 0.01 | 0.03 | 0.01 | 0.03 |
| edu1 [literacy] |  |  | 0.01 | 0.03 | 0.00 | 0.03 |
| householdsize |  |  | -0.01 | 0.00 | -0.01 | 0.00 |
| distanceHZ |  |  | -0.00 | 0.00 | -0.00 | 0.00 |
| SBP1 [≥140] |  |  | -0.04 | 0.03 | -0.04 | 0.03 |
| BMIcal1 [≥25] |  |  | 0.03 | 0.02 | 0.02 | 0.02 |
| arthritis [yes] |  |  | -0.02 | 0.03 | -0.02 | 0.03 |
| heart [yes] |  |  | 0.03 | 0.03 | 0.03 | 0.03 |
| chronic bronchitis [yes] |  |  | -0.11 ^***^ | 0.03 | -0.10 ^***^ | 0.03 |
| insomnia [yes] |  |  | 0.02 | 0.02 | 0.01 | 0.02 |
| smoke1 [Past] |  |  |  |  | 0.05 | 0.05 |
| smoke1 [Current] |  |  |  |  | -0.01 | 0.05 |
| drink1 [Past] |  |  |  |  | 0.02 | 0.05 |
| drink1 [Current] |  |  |  |  | 0.10 | 0.07 |
| R^2^ / R^2^ adjusted | 0.023 / 0.017 | | 0.286 / 0.258 | | 0.291 / 0.258 | |
| ** p<0.05   ** p<0.01   *** p<0.001* | | | | | | |

| **Table Q** Multiple linear regression analyses of religious practices (praying, kowtow, and pilgrimage) on walking endurance (m) among older Amdo Tibetans. | | | | | | |
| --- | --- | --- | --- | --- | --- | --- |
|  | **Crude** | | **Multivariable adjusted** | | **Full adjusted** | |
| *Predictors* | *Beta* | *SE* | *Beta* | *SE* | *Beta* | *SE* |
| (Intercept) | 109.06 ^***^ | 5.33 | 111.11 ^***^ | 9.23 | 108.94 ^***^ | 9.53 |
| pray orNot [yes] | -10.08 ^*^ | 5.43 | -5.72 | 5.18 | -4.30 | 5.26 |
| kowtow orNot [yes] | -6.73 ^**^ | 2.51 | -5.46 ^*^ | 2.43 | -4.93 ^*^ | 2.45 |
| pilgrimage orNot [yes] | -2.16 | 2.37 | -1.63 | 2.30 | -1.76 | 2.30 |
| age |  |  | -0.41 ^***^ | 0.11 | -0.40 ^***^ | 0.11 |
| gender [male] |  |  | 11.24 ^***^ | 2.56 | 8.93 ^**^ | 2.81 |
| yak |  |  | -0.03 | 0.03 | -0.03 | 0.03 |
| expense_yearly120000-34999 |  |  | 7.49 ^**^ | 2.67 | 7.63 ^**^ | 2.68 |
| expense yearly1 [>35000] |  |  | 5.22 | 2.94 | 5.26 | 2.94 |
| siblings |  |  | 0.11 | 0.54 | -0.10 | 0.55 |
| marital status2  [unmarried] |  |  | -7.10 | 7.77 | -8.26 | 7.78 |
| marital status2  [widow/widower] |  |  | -8.89 ^*^ | 3.57 | -8.92 ^*^ | 3.57 |
| edu1 [literacy] |  |  | 3.23 | 2.90 | 2.99 | 2.91 |
| householdsize |  |  | 0.41 | 0.46 | 0.36 | 0.46 |
| distanceHZ |  |  | 0.17 ^***^ | 0.05 | 0.18 ^***^ | 0.05 |
| SBP1 [≥140] |  |  | 0.46 | 2.67 | 0.78 | 2.69 |
| BMIcal1 [≥25] |  |  | -3.40 | 2.26 | -3.29 | 2.26 |
| arthritis [yes] |  |  | 5.06 | 2.68 | 5.14 | 2.68 |
| heart [yes] |  |  | -7.51 ^*^ | 3.33 | -7.23 ^*^ | 3.33 |
| chronic bronchitis [yes] |  |  | -7.09 ^*^ | 3.24 | -6.75 ^*^ | 3.25 |
| insomnia [yes] |  |  | -1.81 | 2.53 | -1.96 | 2.54 |
| smoke1 [Past] |  |  |  |  | 4.60 | 5.40 |
| smoke1 [Current] |  |  |  |  | 9.61 | 5.41 |
| drink1 [Past] |  |  |  |  | 1.79 | 5.15 |
| drink1 [Current] |  |  |  |  | 5.01 | 7.89 |
| R^2^ / R^2^ adjusted | 0.027 / 0.021 | | 0.193 / 0.162 | | 0.201 / 0.164 | |
| ** p<0.05   ** p<0.01   *** p<0.001* | | | | | | |

| **Table R.** Full model of religious practices (pray, kowtow, and pilgrimage) mental health (psychosocial well-being, psychological distress, social integration, and self-rated health), physical activity (moderate-to-vigorous physical activity, sleep duration), and physical function (grip strength, gait speed, and walking endurance) among older Amdo Tibetans. | | | | | | | | | | | | | | | | |
| --- | --- | --- | --- | --- | --- | --- | --- | --- | --- | --- | --- | --- | --- | --- | --- | --- |
|  | **SRS** | **Flourish** | **Optimism** | **Mastery** | **CESD** | **GAD** | **Hopeless** | **F Soz U** | **C KMS** | **SRH** | **MVPA** | **Sleep** | **Grip** | **Gait speed** | **Endurance** |  |
|  | *βSE P* | *βSE P* | *βSE P* | *βSE P* | *βSE P* | *βSE P* | *βSE P* | *βSE P* | *βSE P* | *βSE P* | *βSE P* | *βSE P* | *βSE P* | *βSE P* | *βSE P* |  |
| Pray [yes] | 0.41 | 3.6 | 0.34 | -0.36 | 0.25 | **-2.33 ^**^** | 0.4 | **2.16 ^**^** | **0.70 ^*^** | -0.41 | **-1.36 ^*^** | 0.02 | **-3.09 ^*^** | -0.07 | -4.3 |  |
|  | -0.59 | -2.15 | -0.32 | -0.29 | -0.4 | -0.73 | -0.29 | -0.8 | -0.32 | -0.23 | -0.52 | -0.24 | -1.5 | -0.05 | -5.26 |  |
| Kowtow [yes] | **0.83 ^**^** | **3.27 ^**^** | **0.61 ^***^** | **0.37 ^**^** | **-0.44 ^*^** | -0.22 | **-0.33 ^*^** | 0.14 | -0.18 | **0.54 ^***^** | **0.44 ^*^** | **0.32 ^**^** | **1.87 ^**^** | **0.08 ^***^** | **-4.93 ^*^** |  |
|  | -0.28 | -1 | -0.15 | -0.13 | -0.18 | -0.34 | -0.14 | -0.37 | -0.15 | -0.1 | -0.23 |  | -0.7 | -0.02 | -2.45 |  |
| Pilgrimage [yes] | **-0.64 ^*^** | 1.54 | 0.15 | -0.24 | -0.18 | -0.06 | **-0.37 ^**^** | -0.17 | -0.11 | -0.07 | **-0.52 ^*^** | 0.01 | -0.47 | 0.02 | -1.76 |  |
|  | -0.26 | -0.94 | -0.14 | -0.13 | -0.17 | -0.32 | -0.13 | -0.35 | -0.14 | -0.1 | -0.22 | -0.1 | -0.65 | -0.02 | -2.3 |  |
| Age | -0.02 | **0.11 ^*^** | 0.00 | 0.00 | 0.01 | 0 | **0.01 ^*^** | -0.01 | 0 | 0.01 | **-0.04 ^***^** | **0.02 ^***^** | **-0.30 ^***^** | **-0.01 ^***^** | **-0.40 ^***^** |  |
|  | -0.01 | -0.04 | -0.01 | -0.01 | -0.01 | -0.02 | -0.01 | -0.02 | -0.01 | 0 | -0.01 | -0.01 | -0.03 | 0 | -0.11 |  |
| Sex[male] | **0.89 ^**^** | 0.97 | 0.27 | **0.60 ^***^** | -0.05 | -0.55 | -0.15 | -0.06 | 0.04 | 0.22 | -0.38 | **0.54 ^***^** | **10.34 ^***^** | **0.10 ^***^** | **8.93 ^**^** |  |
|  | -0.32 | -1.15 | -0.17 | -0.15 | -0.21 | -0.39 | -0.16 | -0.43 | -0.17 | -0.12 | -0.27 | -0.13 | -0.8 | -0.03 | -2.81 |  |
| No. yak | 0.00 | 0.00 | 0.00 | 0.00 | 0.00 | 0.01 | 0.00 | 0.00 | 0.00 | 0.00 | **-0.01 ^*^** | 0.00 | 0.00 | 0.00 | -0.03 |  |
|  | 0.00 | -0.01 | 0.00 | 0.00 | 0.00 | 0.00 | 0.00 | 0.00 | 0.00 | 0.00 | 0.00 | 0.00 | -0.01 | 0.00 | -0.03 |  |
| Annual expenditure [20000-34999] | **0.90 ^**^** | **5.03 ^***^** | **0.58 ^***^** | **0.53 ^***^** | **-0.67 ^**^** | **-1.68 ^***^** | -0.11 | **1.00 ^*^** | 0.25 | 0.17 | 0.16 | 0.03 | 1.01 | **-0.10 ^***^** | **7.63 ^**^** |  |
|  | -0.3 | -1.1 | -0.16 | -0.15 | -0.2 | -0.37 | -0.15 | -0.41 | -0.16 | -0.11 | -0.26 | -0.12 | -0.76 | -0.03 | -2.68 |  |
| Annual expenditure [>35000] | **1.44 ^***^** | **5.13 ^***^** | **0.40 ^*^** | **0.54 ^***^** | **-0.54 ^*^** | **-2.22 ^***^** | 0.24 | **1.92 ^***^** | **0.59 ^**^** | 0.08 | **0.59 ^*^** | -0.07 | 1.16 | **-0.07 ^*^** | 5.26 |  |
|  | -0.33 | -1.21 | -0.18 | -0.16 | -0.22 | -0.41 | -0.16 | -0.45 | -0.18 | -0.13 | -0.28 | -0.13 | -0.84 | -0.03 | -2.94 |  |
| No. siblings | -0.07 | 0.32 | 0 | 0.03 | 0.05 | 0.04 | 0.10 ^**^ | -0.09 | 0.01 | 0.02 | -0.08 | 0.03 | 0.2 | 0 | -0.1 |  |
|  | -0.06 | -0.23 | -0.03 | -0.03 | -0.04 | -0.08 | -0.03 | -0.08 | -0.03 | -0.02 | -0.06 | -0.03 | -0.16 | -0.01 | -0.55 |  |
| Marital status | -0.41 | -4.2 | -0.14 | -0.03 | -0.75 | -0.57 | -0.41 | 1.11 | -0.11 | -0.43 | **1.93 ^*^** | 0.5 | **-6.74 ^**^** | -0.13 | -8.26 |  |
| [unmarried] | -0.87 | -3.19 | -0.47 | -0.43 | -0.59 | -1.09 | -0.43 | -1.19 | -0.48 | -0.33 | -0.84 | -0.36 | -2.22 | -0.07 | -7.78 |  |
| [widow/widower] | -0.56 | -2.87 ^*^ | -0.34 | 0.09 | 0.4 | 0.28 | 0.07 | -0.16 | **-0.46 ^*^** | -0.16 | 0.44 | 0.16 | 0.03 | 0.01 | **-8.92 ^*^** |  |
|  | -0.4 | -1.46 | -0.22 | -0.19 | -0.27 | -0.5 | -0.2 | -0.54 | -0.22 | -0.15 | -0.39 | -0.16 | -1.02 | -0.03 | -3.57 |  |
| Education  attaiment [literacy] | **-0.65 ^*^** | -1.76 | **-0.48 ^**^** | 0.19 | 0.02 | 0.66 | -0.07 | -0.6 | -0.16 | -0.12 | 0.12 | 0.06 | 0.35 | 0.00 | 2.99 |  |
|  | -0.33 | -1.19 | -0.18 | -0.16 | -0.22 | -0.41 | -0.16 | -0.44 | -0.18 | -0.12 | -0.28 | -0.13 | -0.83 | -0.03 | -2.91 |  |
| Household size | 0.03 | 0.21 | 0.02 | -0.01 | -0.04 | 0.03 | -0.01 | 0.02 | 0.03 | 0 | -0.02 | 0.03 | -0.07 | -0.01 | 0.36 |  |
|  | -0.05 | -0.19 | -0.03 | -0.03 | -0.03 | -0.06 | -0.03 | -0.07 | -0.03 | -0.02 | -0.04 | -0.02 | -0.13 | 0.00 | -0.46 |  |
| Distance to town | 0.00 | **-0.06 ^**^** | 0.00 | 0.00 | 0.00 | **0.02 ^**^** | 0.00 | 0.00 | **-0.02 ^***^** | **-0.01 ^**^** | 0.00 | 0.00 | **-0.06 ^***^** | 0.00 | **0.18 ^***^** |  |
|  | -0.01 | -0.02 | 0.00 | 0.00 | 0.00 | -0.01 | 0.00 | -0.01 | 0 | 0 | -0.01 | 0.00 | -0.01 | 0.00 | -0.05 |  |
| SBP [≥140] | -0.45 | -0.55 | -0.14 | -0.14 | 0 | 0.56 | 0.09 | 0.29 | 0.32 | -0.02 | -0.19 | -0.06 | 0.33 | -0.04 | 0.78 |  |
|  | -0.3 | -1.1 | -0.16 | -0.15 | -0.2 | -0.38 | -0.15 | -0.41 | -0.16 | -0.12 | -0.28 | -0.12 | -0.77 | -0.03 | -2.69 |  |
| BMI [≥25] | 0.02 | -0.15 | 0.01 | 0.12 | -0.07 | -0.08 | -0.03 | -0.01 | -0.24 | -0.05 | 0.16 | 0.11 | 1.28 ^*^ | 0.02 | -3.29 |  |
|  | -0.25 | -0.92 | -0.14 | -0.12 | -0.17 | -0.32 | -0.13 | -0.34 | -0.14 | -0.1 | -0.22 | -0.1 | -0.64 | -0.02 | -2.26 |  |
| Arthritis [yes] | **-0.85 ^**^** | **-5.36 ^***^** | **-0.43 ^**^** | -0.05 | **0.67 ^**^** | **0.77 ^*^** | 0.29 | **-2.78 ^***^** | **-0.43 ^**^** | **-0.29 ^*^** | 0.22 | -0.16 | -0.12 | -0.02 | 5.14 |  |
|  | -0.3 | -1.1 | -0.16 | -0.15 | -0.2 | -0.37 | -0.15 | -0.41 | -0.16 | -0.12 | -0.27 | -0.12 | -0.77 | -0.03 | -2.68 |  |
| Heart [yes] | -0.69 | **-4.03 ^**^** | -0.02 | -0.36 | **0.73 ^**^** | **1.33 ^**^** | -0.09 | 0.38 | 0.12 | **-0.45 ^**^** | -0.56 | -0.15 | **-3.59 ^***^** | 0.03 | -7.23 ^*^ |  |
|  | -0.37 | -1.36 | -0.2 | -0.18 | -0.25 | -0.47 | -0.19 | -0.51 | -0.2 | -0.14 | -0.31 | -0.15 | -0.95 | -0.03 | -3.33 |  |
| Chronic bronchitis [yes] | -0.36 | 0.16 | 0.24 | 0.21 | -0.43 | **1.37 ^**^** | **-0.40 ^*^** | **1.35 ^**^** | -0.04 | **0.29 ^*^** | -0.38 | 0.13 | -1.42 | **-0.10 ^***^** | **-6.75 ^*^** |  |
|  | -0.37 | -1.33 | -0.2 | -0.18 | -0.25 | -0.45 | -0.18 | -0.5 | -0.2 | -0.14 | -0.34 | -0.15 | -0.93 | -0.03 | -3.25 |  |
| Insomnia [yes] | **-0.93 ^**^** | **-3.44 ^**^** | -0.17 | -0.26 | **1.75 ^***^** | **1.37 ^***^** | -0.04 | -0.43 | -0.22 | **-0.39 ^***^** | **-0.53 ^*^** | -0.2 | 1.14 | 0.01 | -1.96 |  |
|  | -0.29 | -1.04 | -0.15 | -0.14 | -0.19 | -0.35 | -0.14 | -0.39 | -0.16 | -0.11 | -0.25 | -0.12 | -0.72 | -0.02 | -2.54 |  |
| Smoke [Past] | -0.24 | 1.11 | -0.1 | **0.67 ^*^** | 0.23 | -1.03 | 0.45 | -0.4 | 0.06 | 0.02 | -0.04 | -0.4 | -0.01 | 0.05 | 4.6 |  |
|  | -0.61 | -2.21 | -0.33 | -0.3 | -0.41 | -0.75 | -0.3 | -0.82 | -0.33 | -0.23 | -0.5 | -0.25 | -1.54 | -0.05 | -5.4 |  |
| Smoke [Current] | 0.98 | 2.7 | -0.26 | 0.26 | -0.28 | -0.44 | -0.05 | **2.08 ^*^** | **0.74 ^*^** | 0.37 | -0.16 | 0.09 | **5.39 ^***^** | -0.01 | 9.61 |  |
|  | -0.61 | -2.22 | -0.33 | -0.3 | -0.41 | -0.76 | -0.3 | -0.83 | -0.33 | -0.23 | -0.46 | -0.25 | -1.54 | -0.05 | -5.41 |  |
| Drink  [Past] | -0.93 | -2.48 | 0.12 | 0.32 | 0.65 | **2.16 ^**^** | -0.05 | **-1.98 ^*^** | -0.27 | 0.05 | 0.69 | 0.2 | 2.72 | 0.02 | 1.79 |  |
|  | -0.58 | -2.11 | -0.31 | -0.28 | -0.39 | -0.72 | -0.29 | -0.79 | -0.31 | -0.22 | -0.52 | -0.24 | -1.47 | -0.05 | -5.15 |  |
| Drink [Current] | -0.09 | 0.71 | 0.48 | **-1.06 ^*^** | -0.29 | 1.15 | -0.86 | 1.17 | 0.21 | 0.24 | -1.39 | -0.49 | 3.81 | 0.1 | 5.01 |  |
|  | -0.89 | -3.23 | -0.48 | -0.43 | -0.6 | -1.1 | -0.44 | -1.21 | -0.48 | -0.34 | -0.97 | -0.36 | -2.25 | -0.07 | -7.89 |  |
| (Intercept) | 16.33 ^***^ | 69.40 ^***^ | 7.01 ^***^ | 4.42 ^***^ | 1.58 ^*^ | 3.34 ^*^ | 4.58 ^***^ | 23.60 ^***^ | 14.73 ^***^ | 2.60 ^***^ | 8.19 ^***^ | 6.14 ^***^ | 40.82 ^***^ | 1.43 ^***^ | 108.94 ^***^ |  |
|  | -1.07 | -3.9 | -0.58 | -0.52 | -0.72 | -1.33 | -0.53 | -1.46 | -0.58 | -0.41 | -0.98 | -0.44 | -2.72 | -0.09 | -9.53 |  |
| R^2^ / R^2^ adjusted | 0.188 / 0.150 | 0.224 / 0.187 | 0.114 / 0.072 | 0.177 / 0.139 | 0.281 / 0.248 | 0.213 / 0.176 | 0.107 / 0.065 | 0.186 / 0.147 | 0.175 / 0.137 | 0.175 / 0.136 | 0.223 / 0.152 | 0.124 / 0.083 | 0.574 / 0.554 | 0.291 / 0.258 | 0.201 / 0.164 |  |
| ** p<0.05,** p<0.01,*** p<0.001*  SRS: resilience, CESD: depression, GAD: anxiety, F Soz U: social support, C KMS: marital satisfaction, SRH: self-rated health, MHMET: moderate-to-vigorous physical activity duration per day, Sleep: sleep duration per day, Grip: grip strength, Endurance: walking endurance, BMI: body mass index, SBP: systolic blood pressure. | | | | | | | | | | | | | | | | |

**
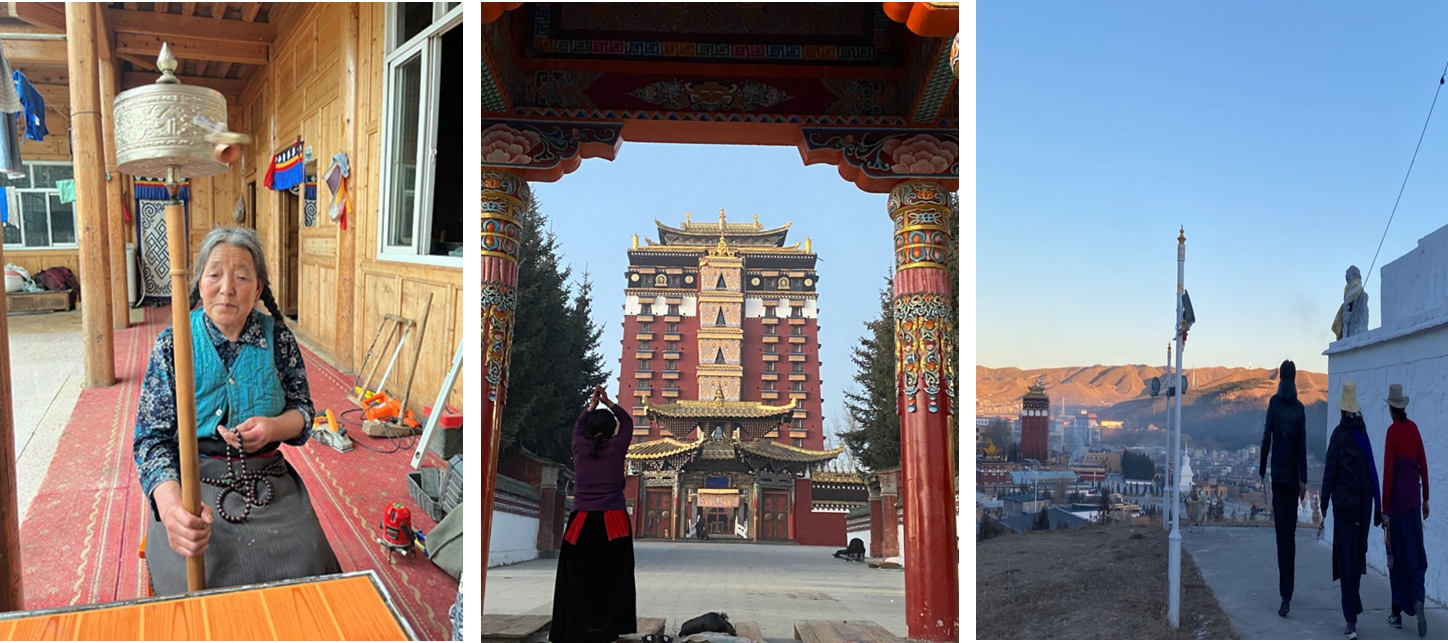
**

**Fig A.** Older Amdo Tibetans participating in religious practices (left panel: pray, middle panel: kowtow, right panel: pilgrimage; Photo credit: Liqiong Zhou)

**References**

1 Zeng Y, Shen K. Resilience Significantly Contributes to Exceptional Longevity. *Curr Gerontol Geriatr Res* 2010; **2010**: 1–9.

2 VanderWeele TJ. On the promotion of human flourishing. *Proc Natl Acad Sci* 2017; **114**: 8148–56.

3 Scheier MF, Carver CS, Bridges MW. Distinguishing optimism from neuroticism (and trait anxiety, self-mastery, and self-esteem): A reevaluation of the Life Orientation Test. *J Pers Soc Psychol* 1994; **67**: 1063–78.

4 Schwarzer R, Bäßler J, Kwiatek P, Schröder K, Zhang JX. The Assessment of Optimistic Self‐beliefs: Comparison of the German, Spanish, and Chinese Versions of the General Self‐efficacy Scale. *Appl Psychol* 1997; **46**: 69–88.

5 Pearlin LI, Schooler C. The structure of coping. *J Health Soc Behav* 1978; **19**: 2–21.

6 Radloff LS. The CES-D Scale: A Self-Report Depression Scale for Research in the General Population. *Appl Psychol Meas* 1977; **1**: 385–401.

7 Spitzer RL, Kroenke K, Williams JBW, Löwe B. A Brief Measure for Assessing Generalized Anxiety Disorder: The GAD-7. *Arch Intern Med* 2006; **166**: 1092.

8 Beck AT, Weissman A, Lester D, Trexler L. The measurement of pessimism: The Hopelessness Scale. *J Consult Clin Psychol* 1974; **42**: 861–5.

9 Smith J, Ryan L, Fisher GG, Sonnega A, Weir D. Psychosocial and Lifestyle Questionnaire 2006 - 2016. Survey Research Center, Institute for Social Research, University of Michigan, 2017.

10 Kliem S, Mößle T, Rehbein F, Hellmann DF, Zenger M, Brähler E. A brief form of the Perceived Social Support Questionnaire (F-SozU) was developed, validated, and standardized. *J Clin Epidemiol* 2015; **68**: 551–62.

11 Schumm WR, Paff-Bergen LA, Hatch RC, *et al.* Concurrent and Discriminant Validity of the Kansas Marital Satisfaction Scale. *J Marriage Fam* 1986; **48**: 381–7.

12 Ferraro KF. Self-Ratings of Health among the Old and the Old-Old. *J Health Soc Behav* 1980; **21**: 377–83.
